# Novel Chiral Ru(II) Complexes as Potential c-myc G-quadruplex DNA Stabilizers Inducing DNA Damage to Suppress Triple-Negative Breast Cancer Progression

**DOI:** 10.3390/ijms24010203

**Published:** 2022-12-22

**Authors:** Chanling Yuan, Zhixiang Wang, Zongtao Wang, Wentao Liu, Guohu Li, Jinlan Meng, Ruzhen Wu, Qiong Wu, Jiacheng Wang, Wenjie Mei

**Affiliations:** 1 School of Pharmacy, Guangdong Pharmaceutical University, Guangzhou 510006, China; 2 Department of Physiology, School of Life Sciences and Biopharmaceutics, Guangdong Pharmaceutical University, Guangzhou 510006, China; 3 Institute of Biological and Medical Engineering, Guangdong Academy of Sciences, Guangzhou 530316, China; 4 Guangdong Engineering Technology Research Centre of Molecular Probe and Biomedicine Imaging, Guangdong Pharmaceutical University, Guangzhou 510006, China

**Keywords:** chiral ruthenium(II) complexes, triple-negative breast cancer, c-myc G-quadruplex DNA

## Abstract

Currently, effective drugs for triple-negative breast cancer (TNBC) are lacking in clinics. c-myc is one of the core members during TNBC tumorigenesis, and G-rich sequences in the promoter region can form a G-quadruplex conformation, indicating that the c-myc inhibitor is a possible strategy to fight cancer. Herein, a series of chiral ruthenium(II) complexes ([Ru(bpy)_2_(DPPZ-R)](ClO_4_)_2_, ***Λ/Δ***−1: R = -H, ***Λ/Δ***−2: R = -Br, ***Λ/Δ***−3: R = -C≡C(C_6_H_4_)NH_2_) were researched based on their interaction with c-myc G-quadruplex DNA. ***Λ***−**3** and ***Δ***−**3** show high affinity and stability to decrease their replication. Additional studies showed that ***Λ***−**3** and ***Δ***−**3** exhibit higher inhibition against different tumor cells than other molecules. ***Δ***−**3** decreases the viability of MDA-MB-231 cells with an IC_50_ of 25.51 μM, which is comparable with that of cisplatin, with an IC_50_ of 25.9 μM. Moreover, ***Δ***−**3** exhibits acceptable cytotoxic activity against MDA-MB-231 cells in a zebrafish xenograft breast cancer model. Further studies suggested that ***Δ***−**3** decreases the viability of MDA-MB-231 cells predominantly through DNA-damage-mediated apoptosis, which may be because ***Δ***−**3** can induce DNA damage. In summary, the results indicate that Ru(II) complexes containing alkinyl groups can be developed as c-myc G-quadruplex DNA binders to block TNBC progression.

## 1. Introduction

Breast cancer is the most frequent malignant tumor in women worldwide. Among the types of breast cancer, triple-negative breast cancer (TNBC) is an aggressive type of tumor with a fast growth rate and high risks of metastasis and recurrence, which has resulted in a lack of targeted therapies when TNBC is estrogen-receptor-negative, progesterone-receptor-negative and human epidermal growth factor receptor 2-negative [1,2]. Recently, the high expressions of c-myc- and myc-dependent gene signatures have been associated with the aggressiveness of TNBCs [3,4]. Considering that c-myc is one of the core members in TNBC tumorigenesis [5], developing a c-myc inhibitor is a possible strategy to fight cancer.

The transcription factor c-myc is a well-known oncoprotein and an intrinsically disordered protein that lacks available drug recognition sites [6]. Searching for small molecule inhibitors that can directly act on the c-myc protein has long been a major problem in drug development worldwide [7,8]. However, a G-rich sequence of the NHEIII 1 element exists in the promoter region of the c-myc gene, which can form a specific secondary structure of a G-quadruplex, preventing the transcription of c-myc through the stabilization of this conformation, and leading to the downregulation of c-myc expression [9]. Research has indicated that different heteroaromatic molecules with large planar structures exhibit powerful binding affinity and remarkable stability to c-myc G-quadruplex DNA, interfering with transcription [10,11]. In 2001, a class of cationic porphyrin derivatives (i.e., TMPyP2, TMPyP3, and TMPyP4) were regarded as c-myc G-quadruplex DNA binders, which can stabilize the G-quadruplex conformation to decrease the transcription of c-myc [12,13]. Moreover, Huang et al. reported a series of quindoline derivatives and isaindigotone derivatives can effectively bind and stabilize the c-myc G-quadruplex DNA to downregulate c-myc transcription, which inhibits different tumor cell proliferations through cell cycle arrest and apoptosis [14,15]. Hu et al. found that some dibenzoquinoxaline derivatives can effectively inhibit Topo1 activity and strongly stabilize the c-myc G-quadruplex to inhibit the proliferation of TNBC cells [16]. Accordingly, this work suggests that this dual-targeting strategy may be effective in cancer therapy. In summary, targeting c-myc G-quadruplex DNA has become a promising anticancer strategy.

Recently, a class of Ru(II) complexes coordinated with heteroaromatic ligands has shown favorable G-quadruplex-DNA-binding properties with remarkable cytotoxicity against tumor cells. Ji et al. demonstrated that two Ru(II) complexes containing DPPZ ligands display great binding ability to telomeric repeat d(AG_3_[T_2_AG_3_]_3_)G-quadruplex DNA and different DNA stabilities [17]. Liu et al. found that a class of dinuclear Ru(II) complexes, especially ([phen]_2_Ru[bpibp]Ru[phen]_2_)(ClO_4_)_4_, can selectively bind to the c-myc G-quadruplex DNA to decrease the expression of the c-myc gene, resulting in liver cancer HepG2 cell apoptosis [18]. Mei et al. reported that a series of Ru(II) complexes containing phenanthroimidazole derivatives with different structural modifications, such as (Ru[phen]_2_[*p*-tFMPIP])(ClO_4_)_2_, can interact with c-myc G-quadruplex DNA and induce the DNA-damage-mediated apoptosis of tumor cells [19]. Moreover, Cardin et al. found that the lambda enantiomer of the chiral DPPZ-based Ru(II) complex *Λ*-(Ru[phen]_2_[qdppz])^2+^ displays enantiospecific G-quadruplex-binding properties [20]. Thus, a chiral DPPZ-based Ru(II) complex could be developed to act as a c-myc G-quadruplex binder to suppress TNBC metastasis, and the potential mechanism should be clarified.

**Scheme 1:**
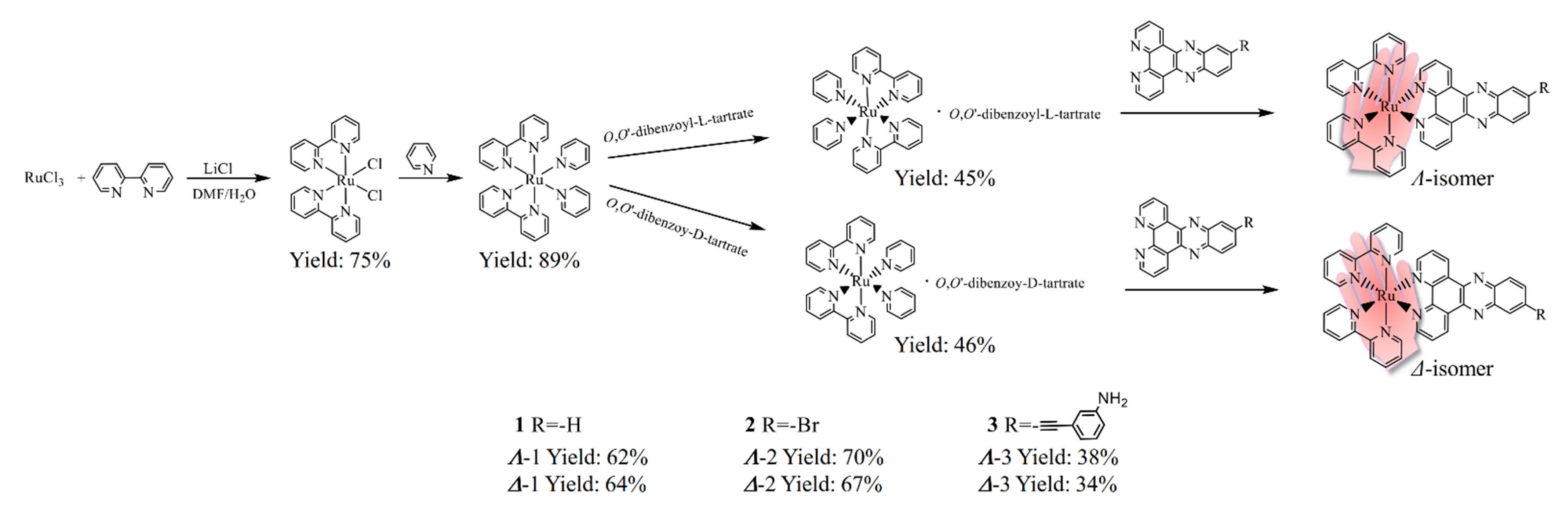
The synthetic route of a class of chiral DPPZ-based ruthenium complexes (***Λ***−**1**−**3** and ***Δ***−**1**−**3**).

In this study, a DPPZ moiety with different substituent modifications in the structure of the chiral polypyridyl Ru(II) complex ([Ru(bpy)_2_(DPPZ-R)](ClO_4_)_2_, ***Λ***−**1** and ***Δ***−**1**: R = -H, ***Λ***−**2** and ***Δ***−**2**: R = -Br, ***Λ***−**3** and ***Δ***−**3**: R = -C≡C(C_6_H_4_)NH_2_) is introduced to improve the planarity of the molecular structure and enhance the affinity to the c-myc G-quadruplex DNA (Scheme 1). Molecular docking and interaction assessments were performed to evaluate the binding properties of the chiral polypyridyl Ru(II) complex with c-myc G-quadruplex DNA. The results show that the introduction of the 3-aminophenylethynyl group is beneficial to the capability to interact with c-myc G-quadruplex DNA and increase the cytotoxicity against breast cancer cells. The analysis of the structure–activity relationship reveals that the introduction of different substituent groups in the terminal benzene ring regulates the planarity and electron cloud density of the molecular structure and improves hydrophilicity or lipophilicity, thereby assisting complexes in entering the cell nucleus. Additional biological studies suggested that the best inhibitory molecule, ***Δ***−**3**, can effectively suppress the proliferation, migration, and invasion of TNBC cells and induce DNA-damage-mediated apoptosis, possibly through the downregulation of c-myc.

## 2. Results and Discussion

### 2.1. Synthesis and Characterization

Some studies have demonstrated that the DPPZ-based Ru(II) complex [Ru(bpy)_2_(DPPZ)](ClO_4_)_2_ exhibits excellent G-quadruplex-DNA-binding ability and can act as molecular “lightswitches” for DNA, but has difficulty penetrating the cell membrane [21,22]. However, our previous research found that the introduction of an electron-rich π-conjugated phenylethynyl group can help the Ru(II) complex enter the tumor cell nucleus [23]. Herein, the 3-aminophenylethynyl group was successfully linked to the terminal benzene ring of [Ru(bpy)_2_(DPPZ-Br)](ClO_4_)_2_ through the Sonogashira coupling reaction under microwave irradiation [24]. Moreover, the levo- and dextro-isomers of [Ru(bpy)_2_(DPPZ-Br)](ClO_4_)_2_ were synthesized using the chiral precursors ([Ru(bpy)_2_(py)_2_][*O,O’*-dibenzoyl-L-tartrate] and [Ru(bpy)_2_(py)_2_][*O,O’*-dibenzoyl-D-tartrate]), and the levo- and dextro-isomers of [Ru(bpy)_2_(3-NBEDPPZ)](ClO_4_)_2_ were obtained through the Sonogashira coupling reaction. The ESI-MS, ^1^H NMR, and ^13^C NMR spectra of these complexes are provided in the Supporting Information (Figures S1–S18). In addition, the CD spectra of ***Λ***−**3** displayed a representative positive CD signal of 134.6 at 290 nm and three negative CD signals at 261, 316, and 420 nm. Symmetrical CD signals of ***Δ***−**3** were also observed in the range of 260–420 nm, with a maximum negative signal at 290 nm (Figure S19).

### 2.2. Structure–Activity Relationship Analysis

The inhibitory effect of the chiral DPPZ-based Ru(II) complexes (***Λ/Δ***−**1**−**3**) was assessed against different human tumor cancer cells, such as breast cancer MCF-7, MDA-MB-231, lung cancer A549, liver carcinoma HepG2, nasopharyngeal carcinoma CNE-1, and human normal keratinocyte HaCaT cells, by MTT assay. The half-maximal inhibitory concentrations (IC_50_) of these synthetic chiral DPPZ-based Ru(II) complexes (***Λ/Δ***−**1**−**3**) and cisplatin against different human tumor cell lines after 72 h of treatment are listed in Table 1.The results reveal that the ***Δ***−**3** complex exhibits acceptable inhibitory effects against MDA-MB-231 cells, with an IC_50_ value of 25.5 μM, which was comparable with that of cisplatin (25.9 μM). However, the inhibitory effect of ***Δ***−**3** against normal human HaCaT cells, with an IC_50_ value of 70.9 μM, was less toxic than that of cisplatin, with an IC_50_ value of 7.1 μM. Moreover, the inhibition of MDA-MB-231 cell proliferation by ***Δ***−**3** was detected by using an EdU (5-ethynyl-2′-deoxyuridine) staining kit, and EdU was incorporated into newly synthesized DNA in the sample cells. The red fluorescent azide is small enough to diffuse freely through native tissues and DNA, and it covalently cross-links to the EdU in a “click” chemistry reaction. As shown in Figure S20, the newly proliferating cells were labelled with red fluorescence by EdU in the control group, with a proliferation rate of 50.2%. However, upon the addition of ***Λ***−**3** and ***Δ***−**3**, the number of newly proliferating cells was markedly reduced. When the concentration of ***Λ***−**3** reached 10 μM and 20 μM, the proliferation rates were 34.0% and 27.5%, respectively, but at 40 μM, the proliferation rate was only 11.3%. However, for ***Δ***−**3** at concentrations of 10 μM and 20 μM, the proliferation rates were 34.9% and 22.0%, respectively, and at 40 μM, the proliferation rate was only 5.1%. Poynton et al. found that chiral Ru(II) complex ***Λ/Δ***-[Ru(phen)_2_(dppz)]^2+^ exhibited great DNA-binding properties but weak inhibitory effect against multiple tumor cells [25]; however, the synthetic chiral Ru(II) complex containing 3-aminophenylethynyl group ***Λ***−**3** and ***Δ***−**3** exhibited greater cytotoxicity against the proliferation of MDA-MB-231 cells. The above results indicate that the dextro-isomers of DPPZ-based Ru(II) complexes containing 3-aminophenylethynyl groups exhibited better inhibition and lower toxicity than cisplatin.

**Figure 1:**
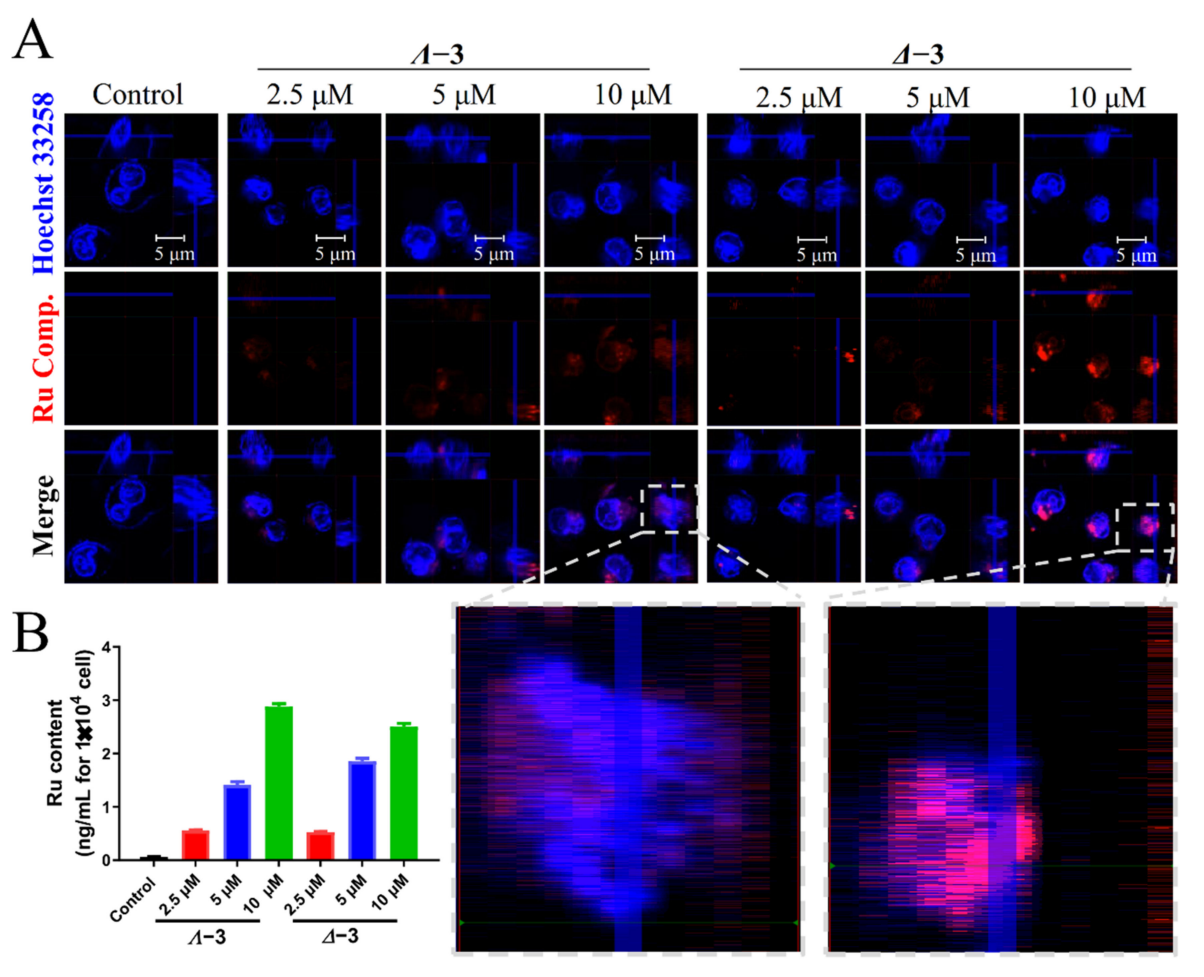
(**A**) The 3D observation of cellular localization for ***Λ***−**3** and ***Δ***−**3** in MDA-MB-231 cells. MDA-MB-231 cells treated with ***Λ***−**3** and ***Δ***−**3** (0, 2.5, 5, and 10 μM) for 24 h. Red: Ru(II) complex, blue: Hoechst33258. (**B**) ICP detection of Ru content in MDA-MB-231 cells treated with different concentrations of Ru(II) complex ***Λ***−**3** and ***Δ***−**3**(0, 2.5, 5, and 10 μM) for 24 h.

Moreover, these chiral DPPZ-based Ru(II) complexes exhibited different inhibitory effects against different tumor cancer cells. The results show that the ***Λ***- and ***Δ***-enantiomers of [Ru(bpy)_2_(DPPZ)](ClO_4_)_2_ exhibited no evident inhibition against various tumor cells due to the impermeable cell properties of this complex [26]. Considering that halogen bonding and the heavy atom effect enhance the electron cloud density and benefit the interaction with potential drug targets, the Br atom was introduced into the terminal benzene ring of [Ru(bpy)_2_(DPPZ)](ClO_4_)_2_ [27,28]. The results show that the presence of Br increased the cytotoxic effect to a certain degree, but the cytotoxic effect was much lower than that of cisplatin. Notably, the introduction of a 3-aminophenylethynyl group helped DPPZ-based Ru(II) complexes effectively penetrate the cell membrane and even cross nuclear membranes. As shown in Figure 1A and Figure S24, the Ru complex (red fluorescence) at 2.5 μM was distributed with the surrounding cell nucleus (blue fluorescence), and some were overlaid with the cell nucleus in the 3D view, indicating that the complex treated with the levo-isomer ***Λ***−**3** (5 μM) in MDA-MB-231 cells could enter the cytoplasm in 24 h. At a concentration of 10 μM, a certain amount of red fluorescent Ru complex was enriched in the nucleus, but the majority of the complex remained abundant in the cytoplasm. However, under the same treatment conditions, the dextro-isomer ***Δ***−**3** at 5 μM could enter the cell nucleus and further increased the enrichment in the cell nucleus at 10 μM in the 3D merge view. These results suggest that the dextro-isomer of the DPPZ-based Ru(II) complex containing a 3-aminophenylethynyl group easily entered the cell nucleus and exhibited a better inhibitory effect than the levo-isomer.

Moreover, as shown in Figure 1B, in the control sample, MDA-MB-231 cells treated without Ru complex, the content of Ru in cell digestion is just 0.4 ng/mL per 1 × 10^4^ cells, but, after treatment with complexes ***Λ***−**3** and ***Δ***−**3**, the content of Ru increased markedly. For complex ***Λ***−**3**, the Ru content is 0.56, 1.44 and 2.47 ng/mL per 1 × 10^4^ cells at 2.5, 5, and 10 μM, respectively. However, for complex ***Δ***−**3**, the Ru content is 0.52, 1.82, and 2.91 ng/mL per 1 × 10^4^ cells at 2.5, 5, and 10 μM, respectively. These results further confirmed that both Ru complexes could enter into cells. The levo-isomer of the PIP-based Ru(II) complex containing a phenylethynyl group more easily entered the cell nucleus than the dextro-isomer, but both isomers exhibited little cytotoxicity against various tumor and normal cells [24]. Li et al. also reported that there is a dramatic difference between the two chiral forms (*Λ-* and *Δ-* enantiomers) of [Ru(bpy)_2_(dppz)]^2+^ and [Ru(phen)_2_(dppz)]^2+^ in live-cell imaging of the DNA nucleus, with Δ-enantiomers showing much brighter emission intensity inside the nucleus than *Λ*-enantiomers [26]. This finding might be because the introduction of a 3-aminophenylethynyl group regulated the enantioselectivity of directional attraction to an electron donor of the DPPZ-based Ru(II) complex.

### 2.3. c-myc G-quadruplex-DNA-Binding Properties

**Figure 2:**
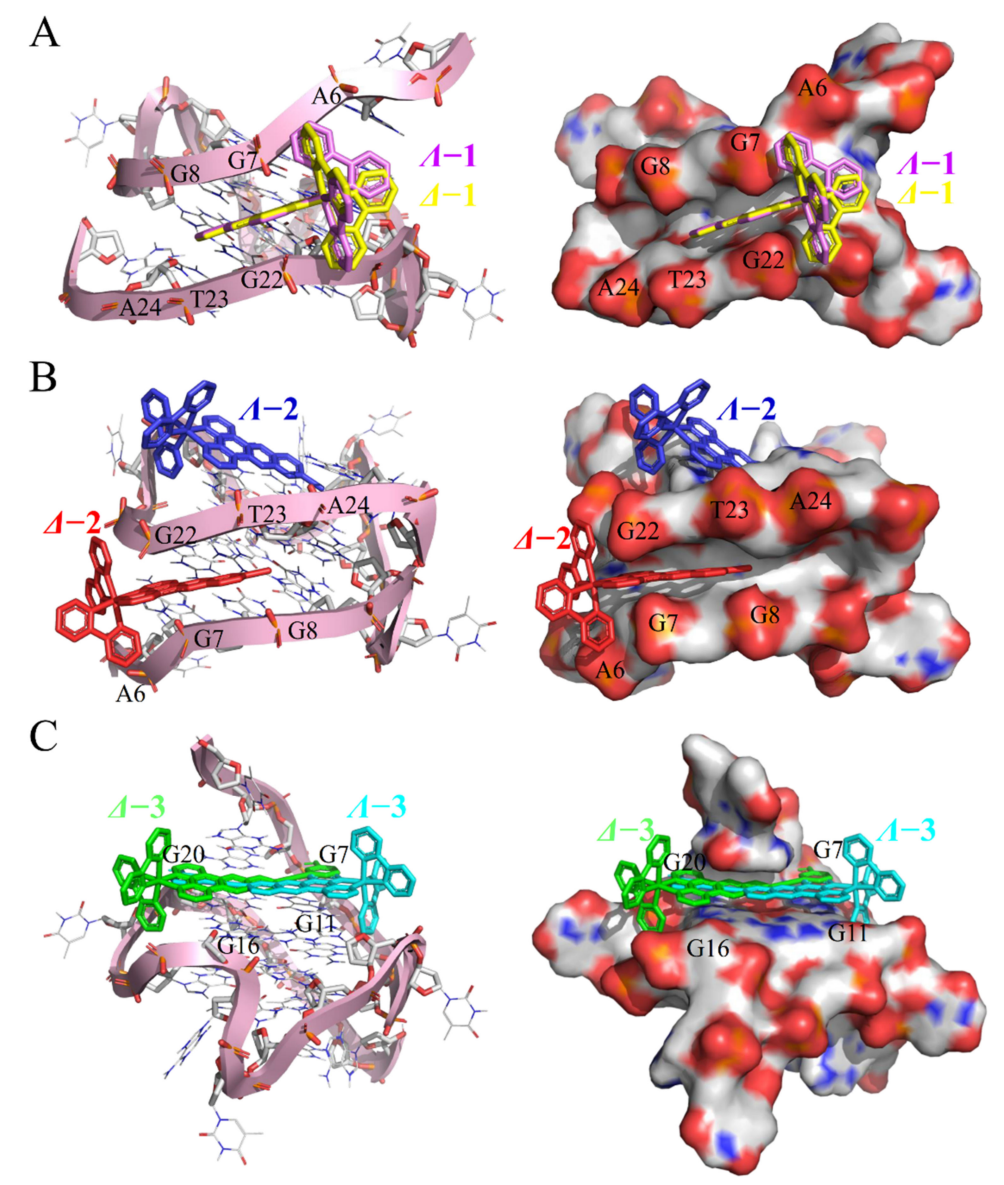
The interaction between chiral DPPZ-based Ru(II)complexes with c-myc G-quadruplex DNA. The binding site and binding mode of (**A**) ***Λ***−**1** (purple) and ***Δ***−**1** (yellow); (**B**) of ***Λ***−**2** (blue) and ***Δ***−**2** (red); and (**C**) of ***Λ***−**3** (cyan) and ***Δ***−**3** (green) with c-myc G-quadruplex DNA calculated by molecular docking.

DNA is a well-known target for metal complexes against tumor cells [29]. A number of studies have shown that G-quadruplex DNA is abundant in the promoter region in various oncogenes [30]. TNBC is accompanied by the apparent amplification of c-myc [31]. Moreover, the DPPZ-based Ru(II) complex exhibits excellent an affinity and stability to c-myc G-quadruplex DNA and reduces transcription to suppress the growth of tumor cells. Herein, molecular docking was used to analyze the interaction of these chiral isomers of the DPPZ-based Ru(II) complex with c-myc G-quadruplex DNA. The results show that ***Λ***−**1** and ***Δ***−**1** of the DPPZ-based Ru(II) complex bound to c-myc G4 DNA through the groove binding mode composed of base pairs of A6, G7, and G8 and G22, T23, and A24 with binding energies of −8.76 and −8.25 kcal·mol^−1^, respectively (Figure 2A). As shown in Figure 2B, ***Λ***−**2** and ***Δ***−**2** of the DPPZ-based Ru(II) complex containing Br atoms exhibited a great affinity to c-myc G4 DNA; however, ***Λ***−**2** interacts with c-myc G4 DNA through a loop binding mode composed of base pairs of G22, T23, and A24 with a binding energy of −8.99 kcal·mol^−1^, and ***Δ***−**2** inserts the groove of base pairs of A6, G7, and G8 and G22, T23, and A24 with a binding energy of −8.74 kcal·mol^−1^. Moreover, the results show that ***Λ***−**3** and ***Δ***−**3** bound to c-myc G4 DNA through the π–π stacking mode but with some differences in directional attraction. As shown in Figure 2C, for ***Λ***−**3**, the large aromatic plane of the 3-NBEDPPZ unit interacted with a planar G-quartet composed of base pairs of G7, G11, G16, and G20 with a binding energy of −9.59 kcal·mol^−1^.Two hydrogen bonds were formed by two H atoms of the 3-amino on the terminal benzene ring interacting with the two O atoms on the G20 residue. Interestingly, for ***Δ***−**3**, the aromatic plane of the 3-NBEDPPZ unit was inserted into the same G-quartet plane in the opposite direction with a binding energy of −9.87 kcal·mol^−1^, which was perfectly symmetrical to that of ***Λ***−**3**. Three hydrogen bonds were formed by N atoms on the pyrazine ring of the ***Δ***−**3** molecule interacting with the H atoms of A6 residues, and the two H atoms on the terminal benzene ring interacted with the two O atoms on the main chain of A6 and G7 residues. These results suggest that the two chiral isomers of the DPPZ-based Ru(II) complex both exhibited a remarkable binding ability to the c-myc G-quadruplex DNA through the π–π stacking mode, but ***Δ***−**3** was stronger, suggesting that the enantioselectivity of the directional attraction is important in regulating the binding properties.

**Figure 3:**
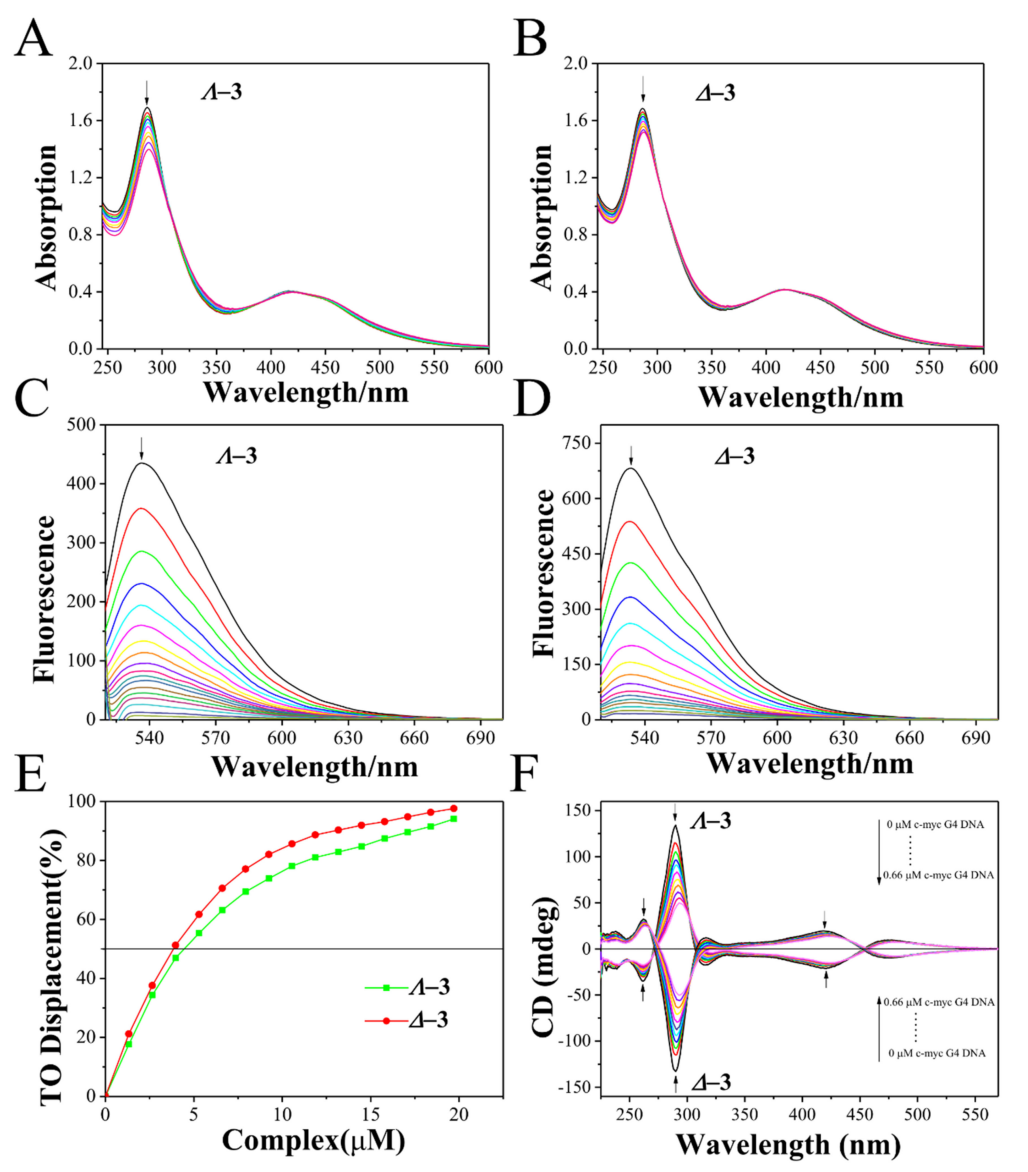
The binding properties of chiral DPPZ-based Ru(II) complexes with c-myc G-quadruplex DNA. The electronic spectra of ***Λ***−**3** (**A**) and ***Δ***−**3** (**B**) at concentration of 20 μM with the increasing of c-myc in the Tris–HCl–KCl buffer. The emission spectra of TO-c-myc system with the increasing of ***Λ***−**3** (**C**) and ***Δ***−**3** (**D**), and (**E**) the DC_50_ values of the relative TO displacement for c-myc G-quadruplex DNA by ***Λ***−**3** and ***Δ***−**3**. [TO] = 1 μM, [Ru] = 0–45 μM, [c-myc] = 0.50 μM, λ_ex_ = 501 nm. (**F**) The CD spectra of ***Λ***−**3** and ***Δ***−**3** upon the addition of c-myc G-quadruplex DNA in Tris-HCl-KCl buffer (pH 7.4).

Herein, studies were performed to evaluate the binding properties and stability of c-myc G-quadruplex DNA interacting with ***Λ***−**3** and ***Δ***−**3** by using different spectroscopic methods. As shown in Figure 3A,B, upon the addition of the c-myc G-quadruplex DNA, the absorption intensities of ***Λ***−**3** and ***Δ***−**3** at a concentration of 20 μM decreased gradually with evident hypochromism of 17.4% and 9.91%, respectively. The intrinsic binding constants (K_b_) of the complex with DNA calculated by the Scatchard equation for ***Λ***−**3** and ***Δ***−**3** were 79.94 and 156.8 × 10^6^ M^−1^, respectively. The above results are in agreement with the study from Svensson et al., who found that the *Δ*-enantiomer of the [Ru(phen)_2_dppz]^2+^ complex has higher DNA-binding activity than the *Λ*-enantiomer [32]. Furthermore, the fluorescence intercalator displacement (FID) assay was used to explore the interaction of ***Λ***−**3** and ***Δ***−**3** with c-myc G-quadruplex DNA. Given the specific high-affinity binding of thiazole orange (TO) to the c-myc G-quadruplex, the DNA exhibited strong fluorescence emission. Additionally, TO dissociated from DNA into the solvent system, thus reducing the fluorescence intensity of the TO-DNA system [33]. With increasing ***Λ***−**3**, a remarkable reduction in the fluorescence intensities of the TO-DNA system with a DC_50_ value of 4.46 μM was observed; for ***Δ***−**3**, the DC_50_ value was 3.84 μM (Figure 3C–E). Moreover, the chiral isomers of DPPZ-based Ru(II) complexes had representative CD signals. The strength of the CD signal decreased with increasing concentrations of the complexes. For ***Λ***−**3**, the strength of the positive CD signal at 290 nm was reduced to 63.1%, whereas the strength of the negative CD signal at 290 nm for ***Δ***−**3** increased to 62.6% (Figure 3F). The results indicate that the chiral isomers of DPPZ-based Ru(II) complexes exhibited remarkable affinity to the c-myc G-quadruplex DNA and that ***Δ***−**3** exhibited slightly stronger binding ability with c-myc G-quadruplex DNA than ***Λ***−**3**. This finding is consistent with the inhibitory effect data.

**Figure 4:**
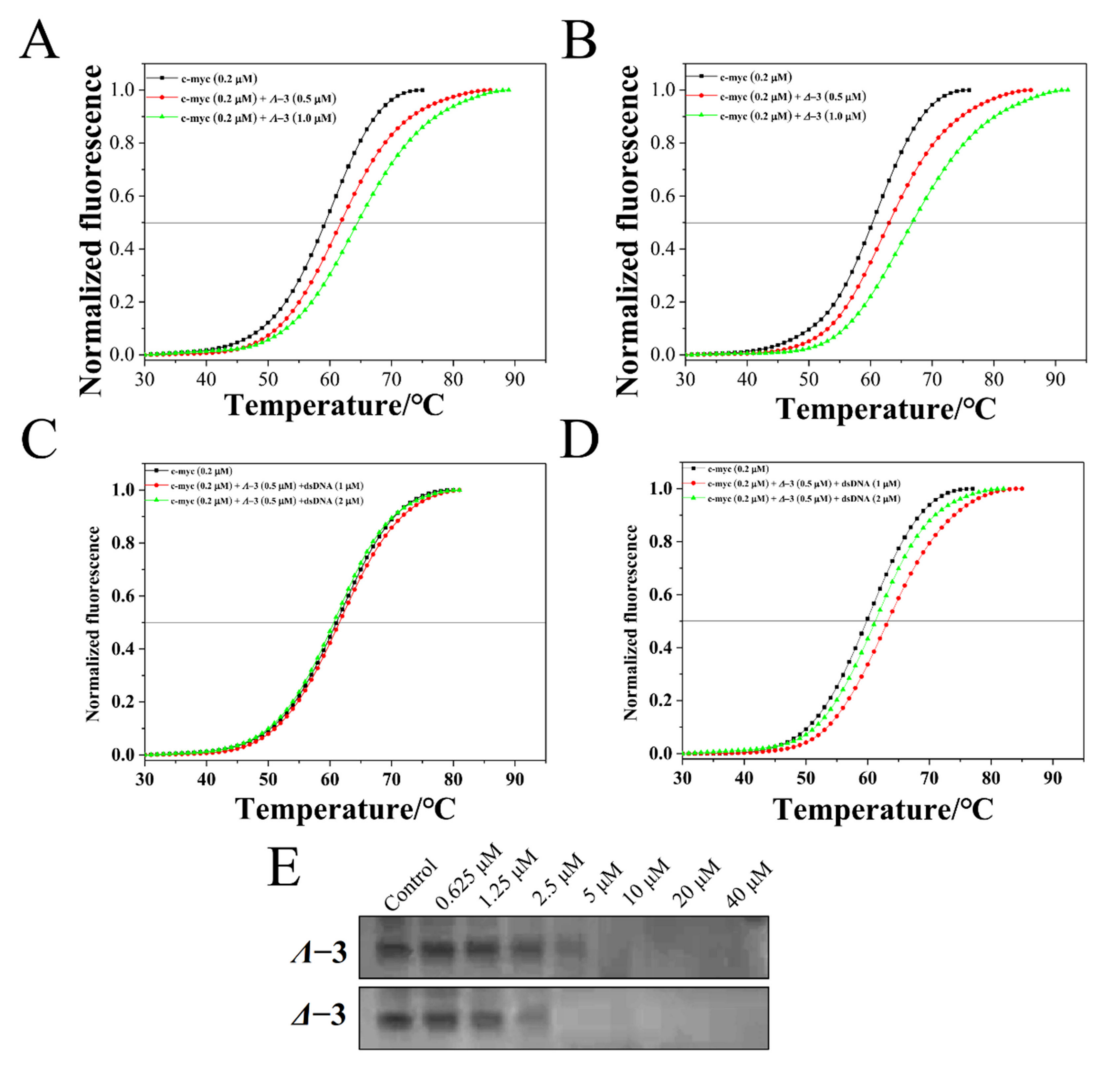
The effect of chiral DPPZ-based Ru(II)complexes on the stability of c-myc G-quadruplex conformation. FRET melting points of c-myc G-quadruplex DNA with different concentrations of ***Λ***−**3** (**A**) and ***Δ***−**3** (**B**). Competitive FRET melting curves of c-myc (0.2 μM) with 0.5 μM of ***Λ***−**3** (**C**) and ***Δ***−**3** (**D**) and a duplex competitor ds26 (1–2 μM). (**E**) Effect of complexes ***Λ***−**3** and ***Δ***−**3** on the PCR-stop assay with c-myc G-quadruplex DNA.

Furthermore, the fluorescence resonance energy transfer (FRET) assay is often utilized to monitor the 3-to-5-end distance to investigate the interaction of ligands with biomacromolecules [34]. FRET melting and competitive FRET assays were performed to investigate the stability of c-myc G-quadruplex DNA induced by the complexes and further confirm the binding ability of ***Λ***−**3** and ***Δ***−**3** with the c-myc G-quadruplex DNA. As shown in Figure 4A,B, upon the addition of ***Λ***−**3** and ***Δ***−**3**, the melting point of the c-myc G-quadruplex DNA notably increased with increasing concentrations of the complexes. The melting point of the free c-myc G-quadruplex DNA was 59.5 °C. When the concentration of ***Λ***−**3** was 0.5 and 1.0 μM, the melting point of the c-myc G-quadruplex DNA significantly increased, with ΔT_m_ values of approximately 2.2°C and 4.9 °C, respectively. When the concentration of ***Δ***−**3** was increased to 0.5 and 1.0 μM, the ΔT_m_ values were approximately 3.5 °C and 7.2 °C, respectively. In FRET competitive experiments (Figure 4C,D), ***Λ***−**3** and ***Δ***−**3** displayed higher selectivity to the c-myc G-quadruplex DNA than to the ds26 duplex DNA. In the presence of excess duplex ds26 DNA, the melting of c-myc G-quadruplex DNA underwent little change. These results suggest that this class of chiral DPPZ-based complexes could stabilize G-quadruplex formation and that ***Δ***−**3** exhibited better stability than ***Λ***−**3**. This finding is in agreement with previous results.

Furthermore, the PCR-stop assay was used to assess the inhibitory effect of complexes against Taq polymerase by stabilizing the c-myc G-quadruplex conformation [35]. As shown in Figure 4E, a significant reduction in the amounts of PCR products was detected as a result of the interaction between Ru(II) complexes and c-myc G-quadruplex DNA. Interestingly, the two chiral isomers showed different inhibitory effects. The minimum concentration of ***Λ***−**3** (5 μM) necessary for effective inhibition with c-myc G-quadruplex DNA was higher than that of the ***Δ***−**3** complex (2.5 μM). This result is comparable with the interaction of c-myc G-quadruplex DNA with other DPPZ-based Ru(II) complexes, which could promote the formation and stabilization of human c-myc G-quadruplex DNA to decrease PCR products. This comparison further reveals that this type of Ru(II) complex could bind to and stabilize the c-myc G-quadruplex DNA to suppress replication. Additionally, ***Δ***−**3** effectively downregulated the expression of the c-myc protein in MDA-MB-231 cells when the concentration reached 40 μΜ (Figure S21). These results indicate that this class of Ru(II) complexes could bind to and stabilize the c-myc G-quadruplex DNA to decrease replication and transcription.

### 2.4. Inhibition of Migration and Invasion of TNBCs

**Figure 5:**
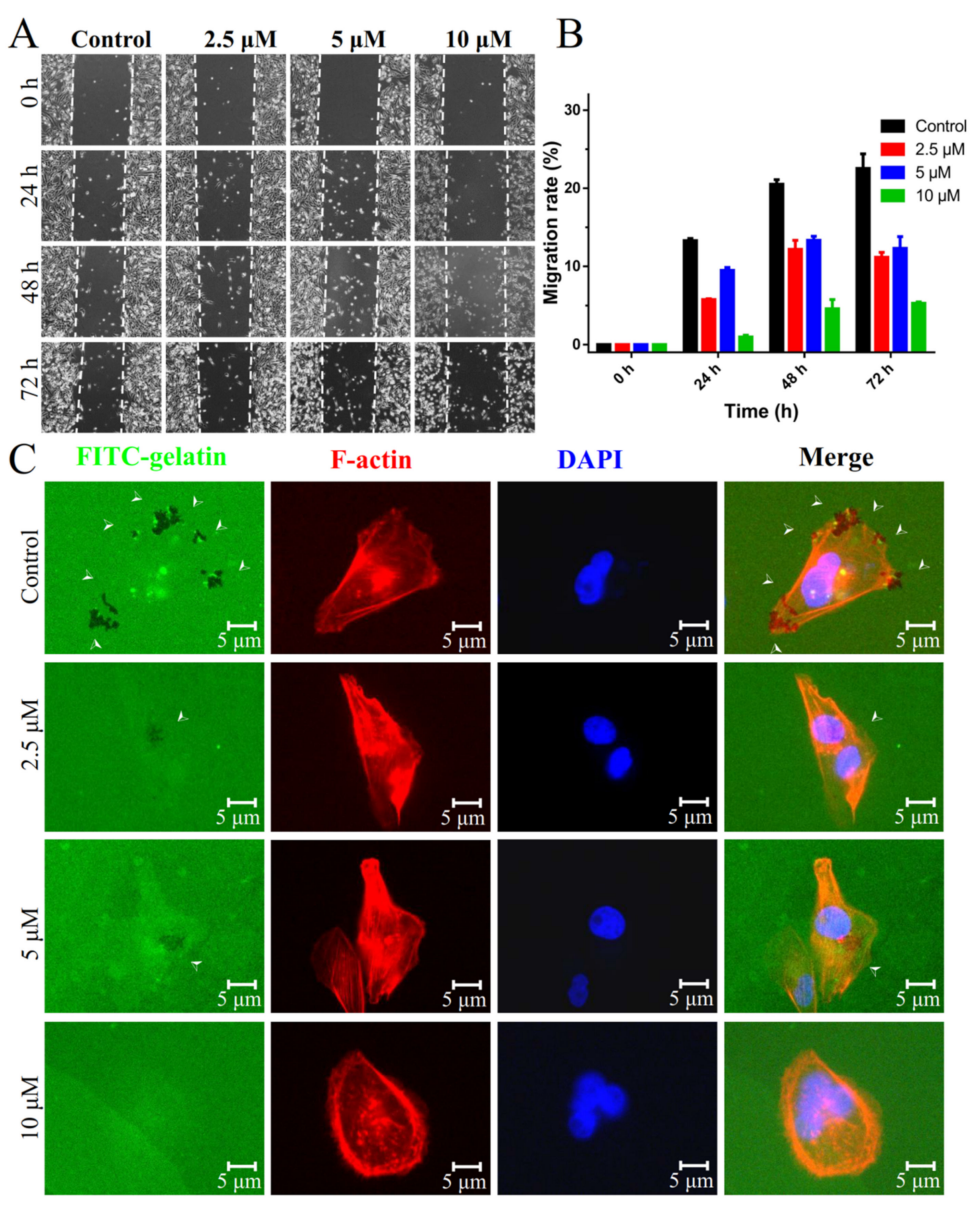
Migration and invasion of MDA-MB-231 cells blocked by ***Δ***−**3** in vitro. (**A**) Wound-healing assay to assess the migration of MDA-MB-231 cells induced by ***Δ***−**3** (0, 2.5, 5, and 10 μM) and DMEM without FBS. The pictures were captured by using a microscope every 24 h.(**B**) Wound-healing rate of MDA-MB-231 cells treated with ***Δ***−**3** (0, 2.5, 5, and 10 μM) (N = 3). (**C**) The FITC–gelatine assay to evaluate the invasive ability of MDA-MB-231 cells was suppressed by ***Δ***−**3** (0, 2.5, 5, and 10 μM). Data were plotted as means ± SEM. Statistical significance was assessed using one-way ANOVA. Values versus the control group: * *p* < 0.05; ** *p* < 0.01; *** *p* < 0.001. ns: no significance.

In clinics, the high metastatic capacity of TNBC is a crucial treatment challenge that has promoted the search for an effective inhibitory agent [36]. To estimate the inhibition of the most active complex, ***Δ***−**3**, against the migration and invasion of MDA-MB-231 cells, a wound-healing assay was applied to assess the MDA-MB-231 cell movement ability induced by different concentrations of ***Δ***−**3** by measuring the distance of scratches. As shown in Figure 5A, without ***Δ***−**3** treatment, the wound closure distance evidently decreased with increasing time. However, the wound-healing distance was restrained upon the addition of ***Δ***−**3**. After treatment with ***Δ***−**3** at 2.5 and 5 μM, the migration of MDA-MD-231 cells was inhibited inconspicuously. However, when the treatment concentration of ***Δ***−**3** was increased to 10 μM, the migration rate was less than half that of the control (Figure 5B) at different treatment times, which indicates that ***Δ***−**3** blocked MDA-MB-231 cell migration in a dose-dependent manner.

Furthermore, the invasive ability of MDA-MB-231 cells inhibited by ***Δ***−**3** was investigated through a FITC–gelatine assay. MDA-MB-231 cells with powerful invasion could excrete large amounts of matrix metalloproteinases to decompose the FITC-labeled gelatine [37]. As shown in Figure 5C, without drug treatment, six degraded areas (black holes) were observed on FITC–gelatine, which overlapped with the MDA-MB-231 cytoskeleton. The results suggest that MDA-MB-231 cells displayed a strong invasive capacity. At ***Δ***−**3** concentrations of 2.5 and 5 μM, the number of degraded areas was markedly reduced. The 10 μM ***Δ***−**3** treatment did not result in a black hole on the FITC–gelatine. The above results reveal that ***Δ***−**3** efficiently suppressed the invasion of MDA-MB-231 cells.

**Figure 6:**
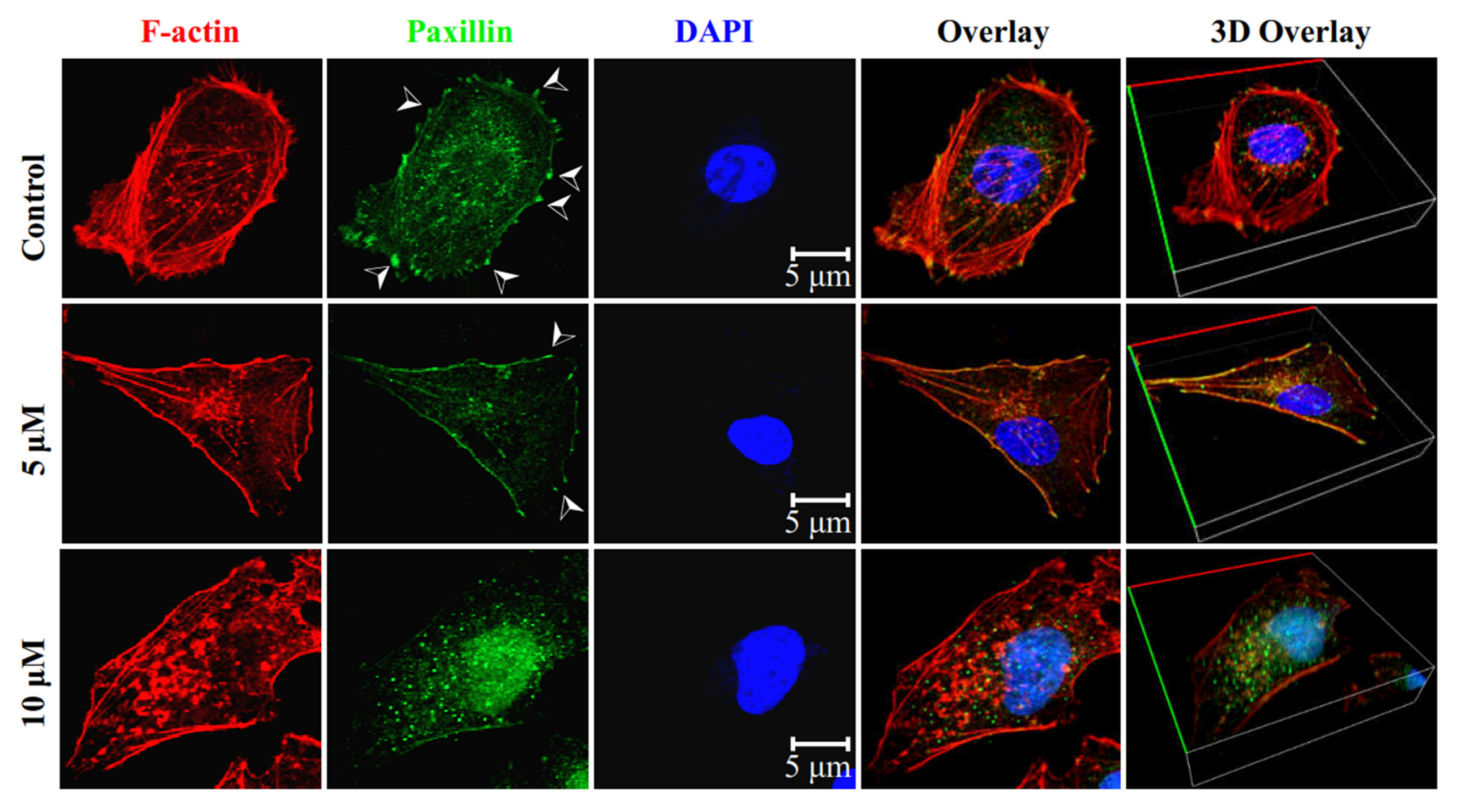
The inhibition of the invadopodia formation of MDA-MB-231 cells induced by ***Δ***−**3**. The immunofluorescence of Paxillin (green) and F-actin (red) in MDA-MB-231 cells after dealt with ***Δ***−**3** (0, 2.5, 5, and 10 μM) for 24 h.

Given that invadopodia are direct executors that invade the extracellular matrix, the formation of invadopodia through the reconstruction of stress fibers and reorganization of the actin cytoskeleton is important for cell migration and invasion [38]. The suppression of stress fibers and focal adhesions in MDA-MB-231 cells by ***Δ***−**3** was investigated by immunofluorescence experiments with F-actin and paxillin. As shown in Figure 6, without ***Δ***−**3** treatment, a number of focal adhesions of paxillin located on the surface of the cell membrane and strong tensile stress fibers of F-actin were observed. However, upon the addition of ***Δ***−**3**, the amount of paxillin decreased, and the cytoskeletal structure became loose. The above results demonstrate that ***Δ***−**3** could block the local adhesion and stress fibers of MDA-MB-231 cells, thus inhibiting their migration and invasion. In summary, this class of DPPZ-based Ru(II) complexes containing 3-aminophenylethynyl could effectively inhibit the proliferation, migration, and invasion of TNBC cells in vitro.

### 2.5. DNA-Damage-Mediated Apoptosis of TNBCs

**Figure 7:**
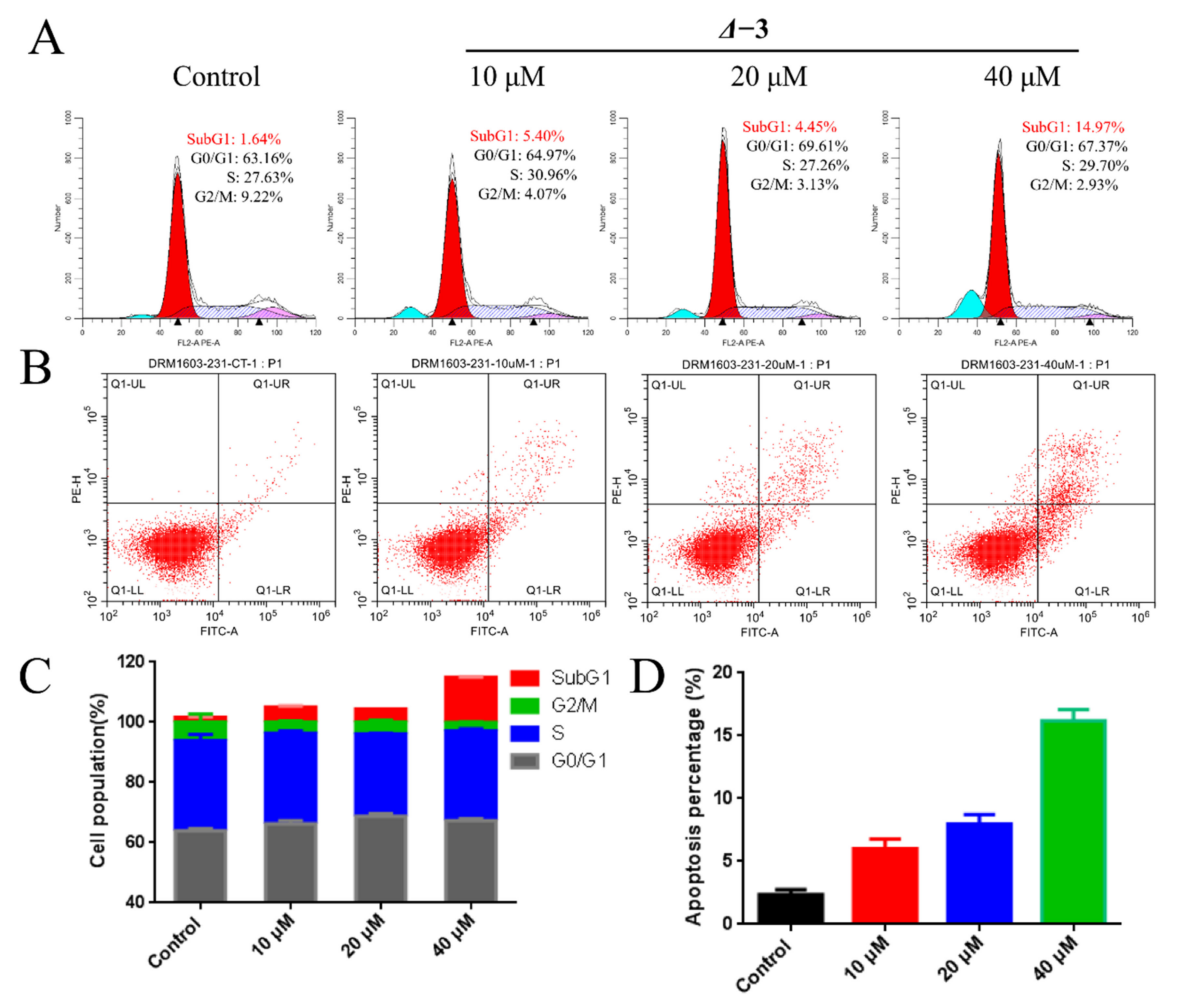
The apoptosis induction of ***Δ***−**3** in MDA-MB-231 cells. (**A**) Cell cycle distribution of MDA-MB-231 cells after treatment with ***Δ***−**3** at different concentrations (0, 10, 20, and 40 μM) for 72 h was detected by flow cytometry. (**B**) Apoptotic rate of MDA-MB-231 cells was measured by flow cytometry after treatment with ***Δ***−**3** (0, 10, 20, and 40 μM) for 72 h. (**C**) Graphical representation of cell-cycle distribution of MDA-MB-231 cells after treatment with ***Δ***−**3**. (**D**) Percentage of the apoptosis in the ***Δ***−**3**-treated MDA-MB-231 cells.

Flow cytometry was used to study the potential inhibitory metastasis mechanism of MDA-MB-231 cells induced by ***Δ***−**3** (Figure S23). As shown in Figure 7A, the treatment of MDA-MB-231 cells with ***Δ***−**3** (0, 10, 20, and 40 μM) for 72 h triggered a weak increase in the number of cells under the G0/G1 phase arrest from 63.16% to 67.37% without evident changes in other cell cycles, which suggests that ***Δ***−**3** had no significant effect on MDA-MB-231 cell cycle regulation. However, upon the addition of ***Δ***−**3**, a certain degree of induction of cell apoptosis was observed. The results show that ***Δ***−**3** notably increased the number of apoptotic cells in a dose-dependent manner. Furthermore, compared with the control with an apoptosis proportion of 2.77%, the proportions of apoptotic cells were 5.39%, 7.95% and 16.27% after 72 h treatment with ***Δ***−**3** at 10, 20, and 40 μM, respectively, revealing that ***Δ***−**3** might suppress the proliferation of TNBC MDA-MB-231 cells by inducing apoptosis. The morphological change as shown in Figure S22.

**Figure 8:**
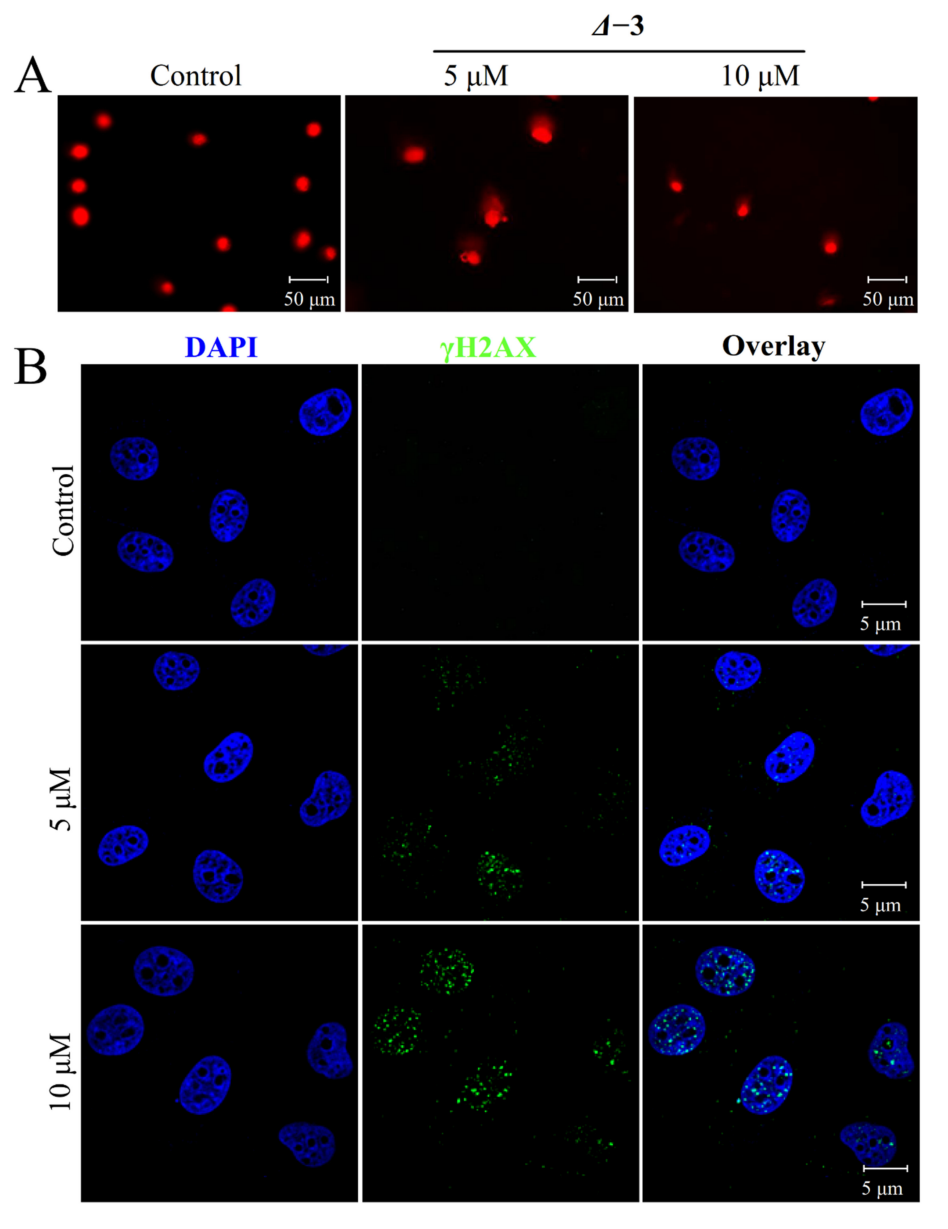
DNA damage induced by ***Δ***−**3**. (**A**) Comet assay to assess the length of tail reflecting DNA damage of MDA-MB-231 cells induced by***Δ***−**3** (0, 5, and 10 μM). (**B**) The expression level of γH2AX in MDA-MB-231 cells treated with ***Δ***−**3** (0, 5, and 10 μM).

The comet assay was used to detect single- or double-stranded DNA breaks in MDA-MB-231 cells induced by ***Δ***−**3**. Cells containing damaged DNA had a comet-like appearance, and the extent of DNA damage could be measured by the length of the tail and the percentage of DNA in the tail. The results are shown in Figure 8A. In the absence of ***Δ***−**3** treatment, MDA-MB-231 cells were round or oval in shape without the cometary phenomenon (control). When treated with ***Δ***−**3** (5 and 10 μM), cometary shapes appeared, suggesting that the complex could induce DNA damage. Given that γ-H2AX is a marker of the DNA damage response, the detection of γ-H2AX protein expression by immunofluorescence assay can be used to further investigate DNA damage. As shown in Figure 8B, no γ-H2AX was produced without ***Δ***−**3** treatment. Green fluorescence was observed after treatment with 5 μM ***Δ***−**3**, demonstrating the production of γ-H2AX and DNA damage. After treatment with 10 μM ***Δ***−**3**, green fluorescence was observed with a distribution wider than that of 5 μM ***Δ***−**3**, indicating that γ-H2AX was produced and that the DNA damage was serious. Thus, we hypothesized that ***Δ***−**3** induced apoptosis in MDA-MB-231 cells by activating DNA damage.

### 2.6. Suppression of Breast Cancer Growth and Metastasis in Vivo

**Figure 9:**
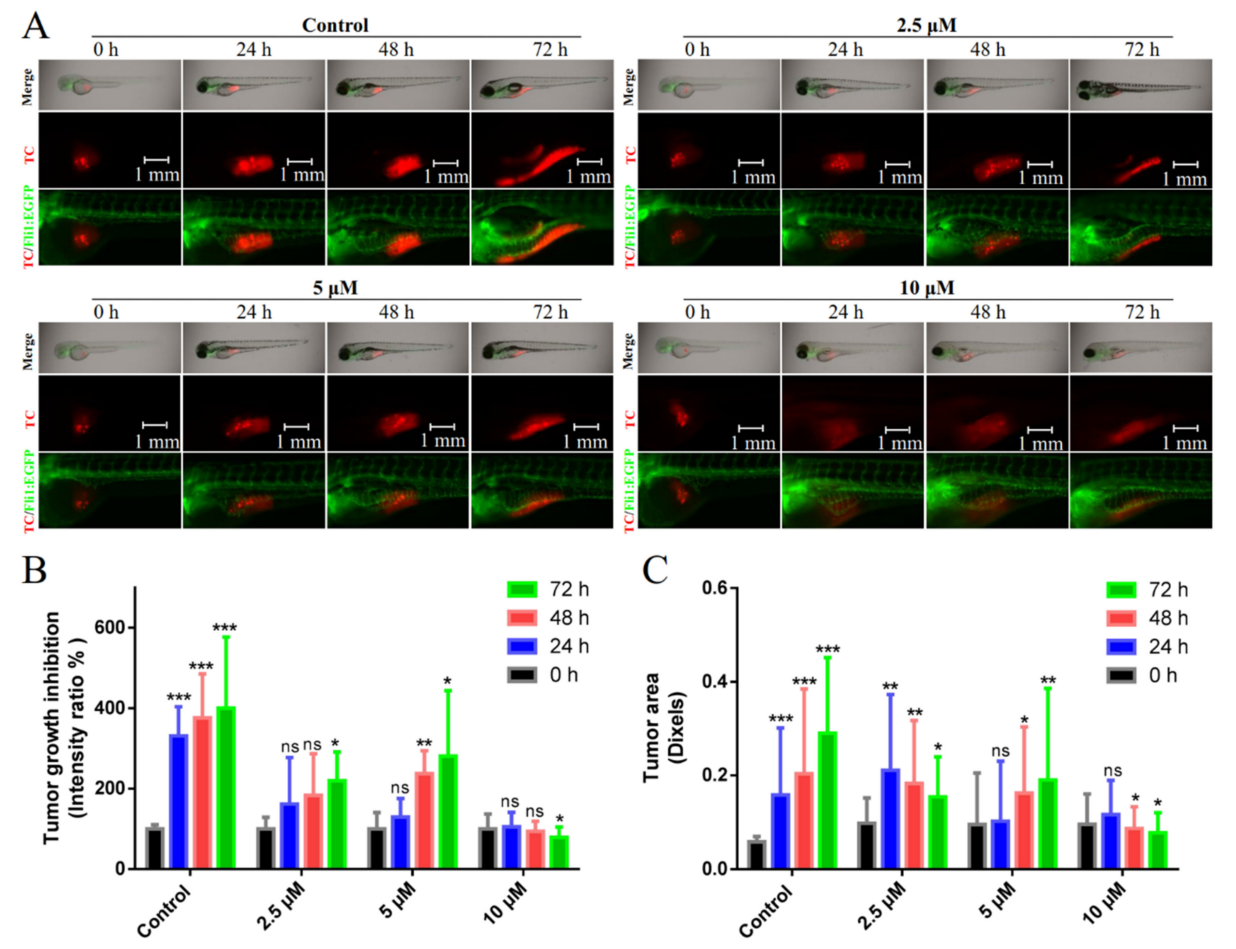
The growth inhibition of MDA-MB-231 cells in zebrafish breast cancer xenografts model inhibited by ***Δ***−**3**. (**A**) The proliferation of MDA-MB-231 cells (red) in breast cancer zebrafish after treatment with ***Δ***−**3** (0, 2.5, 5, and 10 μM) for 72 h. (n = 10/group). The tumor growth inhibition ratio (**B**) and tumor area (**C**) of MDA-MB-231 cells dealt with different concentrations of ***Δ***−**3** in zebrafish. Data were plotted as means ± SD. Statistical significance was assessed using one-way ANOVA. Values versus the control group: * *p* < 0.05; ** *p* < 0.01; *** *p* < 0.001. ns: not significant.

To evaluate the acceptable activity of the ***Δ***−**3** complex against MDA-MB-231 breast cancer cells in vivo, we used an experimental breast cancer xenograft model, which is often used for xenograft tumors in nude mice. We implanted 100–500 fluorescently labeled tumor cells into the blood circulation of zebrafish embryos within 48 h. Blood-borne tumor cells spread within the embryo near the intestinal vessel (SIV) immediately after injection [39]. An extensive main vascular system in the embryo’s yolk sac was observed. As shown in Figure 9A, at 0 h and without drug treatment, few red breast cancer cells spread near the SIV area. At 72 h, MDA-MB-231 cells increased significantly and gathered near the SIV area, indicating that MDA-MB-231 cells could proliferate indefinitely in zebrafish. However, the number of MDA-MB-231 cells after 72 h of treatment with ***Δ***−**3** (2.5, 5, and 10 μM) was significantly lower than that of the control, indicating that ***Δ***−**3** could effectively inhibit the proliferation of zebrafish MDA-MB-231 cells. Hence, ***Δ***−**3** could effectively inhibit the proliferation of MDA-MB-231 cells in vivo and could be developed as a potential candidate for breast cancer inhibition.

### 2.7. Angiogenesis Inhibition in Vitro and in Vivo

**Figure 10:**
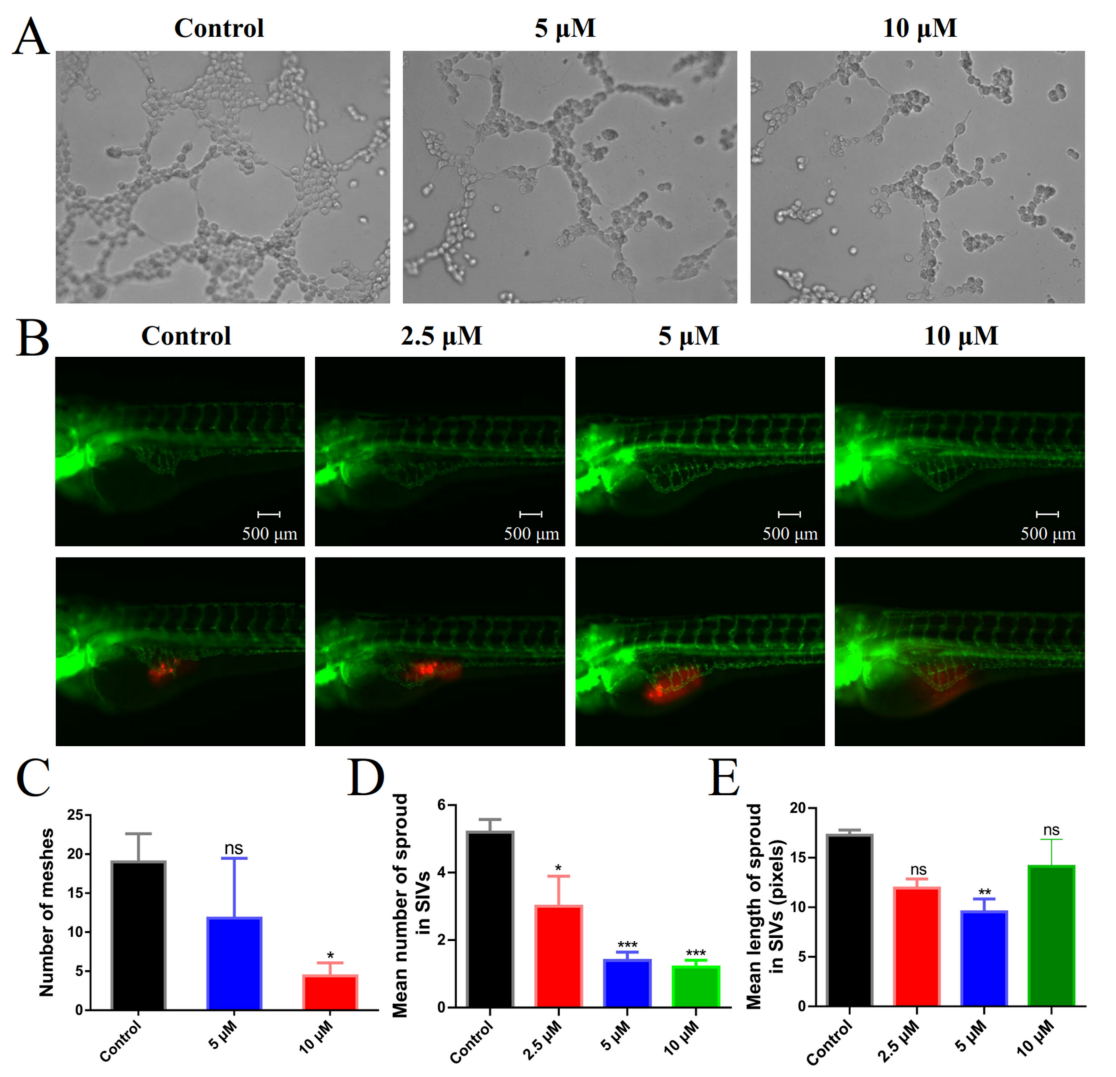
The angiogenesis formation inhibited by ***Δ***−**3** in vitro and in vivo. (**A**) In vitro inhibitory effect of ***Δ***−**3** on the tube formation of HUVECs cells with or without ***Δ***−**3** (0, 5, and 10 μM) for 6 h at 37 °C. The concentration of VEGF is 200 ng/mL. (**B**) Inhibition of angiogenesis in transgenic zebrafish (fil1:EGFP) treated with and without ***Δ***−**3** (0, 2.5, 5, and 10 μM) for 24 h. (**C**) Number of meshes of the tube formation of HUVECs cells (n = 3/group).Data were plotted as means ± SD. Mean number (**D**) and mean length (**E**) of sprouts in SIVs after dealt without and with ***Δ***−**3** as 100% were quantified by manual counting at 72 hpf (n = 5/group). Statistical significance was assessed using one-way ANOVA. Values versus the control group: * *p* < 0.05; ** *p* < 0.01; *** *p* < 0.001. ns: not significant.

Tumor angiogenesis provides large amounts of essential nutrients and oxygen to tumors, maintaining rapid growth and a potential metastatic route [40]. ***Δ***−**3** can block the proliferation of MDA-MB-231 cells in the zebrafish xenograft model. Considering the complicated process of angiogenesis and the formation of an interconnected tubular network by endothelial cells at later stages, the inhibition of the formation of capillary-like tube networks can stop the development of new blood vessels. Therefore, the tube formation assay was adopted by coating HUVECs on Matrigel. HUVECs displayed high mobility on the Matrigel and formed an intact tubular network in the control group. However, when treated with the indicated concentration of ***Δ***−**3**, the ability to form a tubular network was decreased (Figure 10A,C). The intact tubular structures disrupted by 10 μM ***Δ***−**3** were sparse and incomplete. Moreover, the areas of the SIV plexus were smaller and more irregular than those of the control. This result indicates that ***Δ***−**3** could inhibit angiogenesis in vitro. The angiogenic assay was performed in Tg (fli1: EGFP) zebrafish to further assess the antiangiogenic ability of ***Δ***−**3** in vivo (Figure 10B). The findings show that the number and length of SIVs in the ***Δ***−**3** treatment group significantly decreased compared with that in the control group (Figure 10D,E). Overall, compound ***Δ***−**3** might inhibit tumor-cell-induced angiogenesis by binding and stabilizing c-myc G-quadruplex DNA to reduce transcription and expression.

## 3. Materials and Methods

### 3.1. Reagents and Materials

All reagents and solvents are commercially available and were used directly without further treatment unless otherwise specified. The solvent used in all experiments was distilled water. Complexes were synthesized in an Anton Paarmonowave 300 microwave reactor (Anton Paar GmbH, Shanghai, China) using a quartz tube (3.0 mL) with a diameter of 1 cm. The electronic spectrum was recorded on a Shimadzu UV-2550 spectrophotometer. CD spectra were recorded on a Jasco J-810 circular two-color spectrophotometer (JASCO Corporation, Tokyo, Japan). Cell images were collected by fluorescence microscopy (Leica DMI8) and laser-focusing fluorescence microscopy (Zeiss LSM880). The liver cancer HepG2, lung cancer A549, breast cancer MCF-7 and MDA-MB-231, nasopharyngeal carcinoma CNE-1, and keratinocyte HaCaT cells were cultured in DMEM supplemented with 10% foetal bovine serum and 1% penicillin streptomycin.

### 3.2. Synthesis

#### 3.2.1. Synthesis and Characterization of Λ-[Ru(bpy)_2_(DPPZ)](ClO_4_)_2_ (***Λ***−**1**)

Approximately 85 mg of DPPZ (0.3 mmol), 120 mg of *Λ*-[Ru(bpy)_2_(py)_2_][*O,O′*-dibenzoyl-*Λ*-tartrate]·12H_2_O, and ethylene glycol and water mixed solvent (V_glycol_:V_water_ = 9:1) were mixed. After mixing, the crude product was obtained by microwave heating at 120 °C, dissolved in a small amount of acetonitrile, and purified by column chromatography. V_Acetonitrile_:V_toluene_ = 2:1 was used for rinsing. The main red band was washed, and the brick-red solid was obtained by decompression and drying [24]. The yield was 62%. ESI-MS (in CH_3_CN, *m*/*z*): 348.9 ([M-2ClO_4_^−^]^2+^), 795.2 ([M-ClO_4_^−^]^+^).^1^H NMR (600 MHz, DMSO) δ 9.62 (dd, *J* = 8.2, 1.3 Hz, 2H), 8.88 (dd, *J* = 17.4, 8.2 Hz, 4H), 8.52 (dd, *J* = 6.5, 3.4 Hz, 2H), 8.25 (ddd, *J* = 9.4, 6.7, 1.4 Hz, 4H), 8.20 (dd, *J* = 6.5, 3.4 Hz,2H), 8.15 (td, *J* = 8.0, 1.4 Hz, 2H), 8.03 (dd, *J* = 8.2, 5.4 Hz, 2H), 7.85 (dd, *J* = 5.5, 0.5 Hz, 2H), 7.79 (dd, *J* = 5.7, 0.7 Hz, 2H), 7.63–7.60 (m, 2H), 7.40 (ddd, *J* = 7.3, 5.8, 1.2 Hz, 2H). ^13^C NMR (151 MHz, DMSO) δ 155.13 (s), 154.85 (s), 151.79 (s), 150.29 (s), 149.76 (s), 148.68 (s), 140.27 (s), 138.48 (s), 136.44 (s), 136.33 (s), 131.59 (s), 130.94 (s), 128.54 (s), 127.78 (s), 126.28 (s), 126.08 (s), 126.03 (s), 122.87 (s), 122.76 (s).

#### 3.2.2. Synthesis and Characterization of Δ-[Ru(bpy)_2_(DPPZ)](ClO_4_)_2_ (***Δ***−**1**)

Except for the replacement of *Λ*-[Ru(bpy)_2_(py)_2_][*O,O′*-dibenzoyl-*Λ*-tartrate]·12H_2_O with *Δ*-[Ru(bpy)_2_(py)_2_][*O,O′*-dibenzoyl-*Λ*-tartrate]·12H_2_O, the residual preparation method was similar to that of *Λ*-[Ru(bpy)_2_(py)_2_][*O,O′*-dibenzoyl-*Λ*-tartrate]·12H_2_O, with a yield of 64%. ESI-MS (in CH_3_CN, *m*/*z*): 348.3 ([M-2ClO_4_^−^]^2+^), 795.0 ([M-ClO_4_^−^]^+^).^1^H NMR (600 MHz, DMSO) δ 9.64–9.61 (m, 2H), 8.88 (dd, *J* = 17.5, 8.2 Hz, 4H), 8.54–8.50 (m, 2H), 8.24 (ddd, *J* = 9.4, 6.7, 1.4 Hz, 4H), 8.20 (dd, *J* = 6.5, 3.4 Hz, 2H), 8.15 (td, *J* = 8.0, 1.4 Hz, 2H), 8.03 (dd, *J* = 8.2, 5.4 Hz, 2H), 7.86–7.83 (m, 2H), 7.80–7.78 (m, 2H), 7.61 (ddd, *J* = 7.5, 5.6, 1.3 Hz, 2H), 7.40 (ddd, *J* = 7.3, 5.8, 1.2 Hz, 2H). ^13^C NMR (151 MHz, DMSO) δ 157.26 (s), 156.97 (s), 153.91 (s), 152.42 (s), 151.89 (s), 150.81 (s), 142.40 (s), 140.62 (s), 138.57 (s), 138.46 (s), 133.73 (s), 133.08 (s), 130.67 (s), 129.91 (s), 128.41 (s), 128.21 (s), 128.16 (s), 124.99 (s), 124.88 (s).

#### 3.2.3. Synthesis and Characterization of Λ-[Ru(bpy)_2_(BrDPPZ)](ClO_4_)_2_ (***Λ***−**2**)

In addition to the substitution of DPPZ by BrDPPZ (108 mg, 0.3 mmol), the preparation method was similar to that of *Λ*-[Ru(bpy)_2_(DPPZ)](ClO_4_)^2^, with a yield of 70%. ESI-MS (in CH_3_CN, *m*/*z*): 386.7 ([M-2ClO_4_^−^]^2+^), 874.6 ([M-ClO_4_^−^]^+^). ^1^H NMR (500 MHz, DMSO) δ 9.56 (ddd, *J* = 14.6, 8.2, 1.2 Hz, 2H), 8.92–8.85 (m, 4H), 8.70 (d, *J* = 2.2 Hz, 1H), 8.44 (d, *J* = 9.1 Hz, 1H), 8.27 (tdd, *J* = 9.2, 8.6, 1.8 Hz, 5H), 8.16 (tdd, *J* = 8.1, 2.8, 1.4 Hz, 2H), 8.03 (ddd, *J* = 8.2, 6.3, 5.5 Hz, 2H), 7.85 (d, *J* = 5.5 Hz, 2H), 7.82–7.77 (m, 2H), 7.64–7.60 (m, 2H), 7.41 (ddd, *J* = 13.2, 5.7, 1.2 Hz, 2H).^13^C NMR (126 MHz, DMSO) δ 157.25 (s), 156.99 (s), 154.25 (s), 154.11 (s), 152.44 (s), 151.90 (s), 151.03 (s), 150.87 (s), 142.72 (s), 141.28 (s), 141.17 (s), 140.91 (s), 138.60 (s), 138.49 (s), 136.14 (s), 133.76 (s), 131.71 (s), 130.46 (s), 130.37 (s), 128.42 (s), 128.26 (s), 128.22 (s), 126.44 (s), 125.01 (s), 124.91 (s).

#### 3.2.4. Synthesis and Characterization of Δ-[Ru(bpy)_2_(BrDPPZ)](ClO_4_)_2_ (***Δ***−**2**)

Except that BrDPPZ (108 mg, 0.3 mmol) was substituted for DPPZ, the preparation method was similar to that of *Δ*-[Ru(bpy)_2_(DPPZ)](ClO_4_)_2_, with a yield of 67%. ESI-MS (in CH_3_CN, *m*/*z*): 387.1 ([M-2ClO_4_^−^]^2+^), 875.0 ([M-ClO_4_^−^]^+^). ^1^H NMR (500 MHz, *d^6^*-DMSO) δ 9.59 (ddd, *J* = 14.6, 7.3, 3.2 Hz, 2H), 8.89 (dd, *J* = 14.4, 8.2 Hz, 4H), 8.75 (dt, *J* = 3.3, 1.9 Hz, 1H), 8.46 (dd, *J* = 9.1, 2.8 Hz, 1H), 8.27 (dddd, *J* = 15.9, 9.3, 8.5, 1.7 Hz, 5H), 8.18–8.12 (m, 2H), 8.07–8.00 (m, 2H), 7.85 (d, *J* = 5.5 Hz, 2H), 7.79 (t, *J* = 6.5 Hz, 2H), 7.64–7.60 (m, 2H), 7.43–7.39 (m, 2H). ^13^C NMR (126 MHz, *d^6^*-DMSO) δ 157.25 (s), 156.97 (s), 154.24 (s), 154.11 (s), 152.41 (s), 151.89 (s), 151.05 (s), 150.89 (s), 142.75 (s), 141.32 (s), 141.20 (s), 140.96 (s), 138.59 (s), 138.48 (s), 136.14 (s), 133.78 (s), 131.70 (s), 130.49 (s), 130.40 (s), 128.42 (s), 128.27 (s), 128.21 (s), 126.45 (s), 125.00 (s), 124.90 (s).

#### 3.2.5. Synthesis and Characterization of Λ-[Ru(bpy)_2_(3-NBEDPPZ)](ClO_4_)_2_ (***Λ***−−**3**)

*Λ*-[Ru(bpy)_2_(BrDPPZ)]^2+^ (130 mg, 0.125 mmol) was added to a 10 mL microwave reaction tube (with an agitator), and 5 mL of anhydrate acetonitrile was added to the ultrasonic solution until completely dissolved. Under the protection of nitrogen, appropriate amounts of triethylamine and 3-aminobenzene acetylene were added, followed by the rapid addition of palladium copper. After sealing, it was put into a microwave reactor and heated at 140 °C for 30 min [24]. In the column chromatography, pure acetonitrile was used for elution, and three bands were obtained to purify the compound. The acetonitrile:methanol = 30:1 elution collected two bands, which were the target bands. The crude product was a reddish-brown solid with a yield of 38%. ESI-MS (in CH_3_CN, *m*/*z*): 404.9 ([M-2ClO_4_^−^]^2+^), 936.8 ([M-ClO_4_^2-^+I^-^]^+^). ^1^H NMR (500 MHz, *d^6^*-DMSO) δ 9.57 (dd, *J* = 7.6, 5.4 Hz, 2H), 8.90 (dd, *J* = 13.9, 7.6 Hz, 4H), 8.54 (d, *J* = 1.5 Hz, 1H), 8.49 (d, *J* = 8.8 Hz, 1H), 8.29–8.19 (m, 5H), 8.16 (t, *J* = 7.8 Hz, 2H), 8.05 (td, *J* = 8.0, 5.5 Hz, 2H), 7.85 (d, *J* = 5.5 Hz, 2H), 7.81 (dd, *J* = 11.5, 5.6 Hz, 2H), 7.66–7.59 (m, 2H), 7.45–7.39 (m, 2H), 7.15 (t, *J* = 7.8 Hz, 1H), 6.87 (s, 1H), 6.83 (d, *J* = 7.5 Hz, 1H), 6.71 (dd, *J* = 8.2, 1.8 Hz, 1H), 5.38 (s, 2H). ^13^C NMR (126 MHz, *d^6^*-DMSO) δ 156.73 (s), 156.47 (s), 153.73 (s), 153.64 (s), 151.93 (s), 151.40 (s), 150.39 (s), 150.30 (s), 148.99 (s), 141.69 (s), 141.48 (s), 140.85 (s), 140.21 (s), 138.09 (s), 137.99 (s), 134.56 (s), 133.23 (s), 131.48 (s), 130.01 (s), 129.96 (s), 129.41 (s), 127.94 (s), 127.76 (s), 126.50 (s), 124.53 (s), 124.43 (s), 121.62 (s), 119.22 (s), 116.27 (s), 115.46 (s), 95.31 (s), 87.14 (s).

#### 3.2.6. Synthesis and Characterization of Δ-[Ru(bpy)_2_(3-NBEDPPZ)](ClO_4_)_2_ (***Δ***−−**3**)

The synthesis method referred to the synthesis of *Λ*-[Ru(bpy)_2_(3-NBEDPPZ)](ClO_4_)_2_. The yield of crude *Δ*-[Ru(bpy)_2_(BrDPPZ)]^2+^ (130 mg, 0.125 mmol) was 34%. ESI-MS (in CH_3_CN, *m*/*z*): 405.3([M-2ClO_4_^−^]^2+^). ^1^H NMR (500 MHz, *d^6^*-DMSO) δ 9.59–9.54 (m, 2H), 8.91–8.85 (m, 4H), 8.55 (d, *J* = 13.5 Hz, 1H), 8.47 (dd, *J* = 8.8, 4.3 Hz, 1H), 8.27–8.17 (m, 5H), 8.14 (dd, *J* = 12.5, 4.7 Hz, 2H), 8.06–8.00 (m, 2H), 7.83 (d, *J* = 5.6 Hz, 2H), 7.78 (t, *J* = 6.4 Hz, 2H), 7.62–7.58 (m, 2H), 7.40 (t, *J* = 6.6 Hz, 2H), 7.12 (t, *J* = 7.8 Hz, 1H), 6.85–6.84 (m, 1H), 6.81 (d, *J* = 7.5 Hz, 1H), 6.68 (dd, *J* = 8.6, 1.8 Hz, 1H), 5.36 (s, 2H). ^13^C NMR (126 MHz, *d^6^*-DMSO) δ 156.77 (s), 156.50 (s), 153.66 (s), 153.60 (s), 151.97 (s), 151.43 (s), 150.44 (s), 150.35 (s), 149.01 (s), 141.74 (s), 141.52 (s), 140.90 (s), 140.27 (s), 138.11 (s), 138.02 (s), 134.59 (s), 133.28 (s), 131.53 (s), 130.01 (s), 129.44 (s), 127.88 (s), 126.54 (s), 124.56 (s), 124.45 (s), 121.66 (s), 119.26 (s), 116.30 (s), 115.49 (s), 95.34 (s), 87.18 (s).

### 3.3. MTT Assay

Liver cancer HepG2 cells, lung cancer A549 cells, breast cancer MCF-7 and MDA-MB-231 cells, human nasopharyngeal carcinoma CNE-1 cells, and human immortalized keratinocyte HaCaT cells were seeded in 96-well plates (5 × 10^3^ cells per well) and cultured at 37 °C and 5% CO_2_ for 24 h. Cells were treated with different concentrations of the complexes (0, 1.5625, 3.125, 6.25, 12.5, 25, 50, and 100 µM) for 72 h. After adding 20 µL MTT (5 mg/mL) solution to each well, the cells were incubated for 4 h, and then 150 µL DMSO was added to each well. The optical density of each well was detected by a microplate reader at a wavelength of 570 nm. The cell survival rate (%) = A_(experimental group)_/A_(control group)_ × 100%, and the IC_50_ value was analyzed and calculated based on the survival rate.

### 3.4. Cellular Localization

MDA-MB-231(5 × 10^4^ cells/dish) cells were seeded into confocal microscopy dishes [41] and incubated for 24 h at 5% CO_2_. Then, the old culture solution was discarded and the MDA-MB-231 cells were treated with different concentrations of the complex (0, 2.5, 5, and 10 μM) at 37 °C for 24 h. The cell nuclei were stained with Hoechst 33258 for 30 min, and then the cells were washed with PBS three times. This class of Ru complexes emitted red fluorescence with a peak at 580 nm with 405 nm excitation. The cellular localization of the cells was captured using confocal microscopy (Zeiss, LSM880).

### 3.5. ICP Detection

MDA-MB-231 cells were seeded in 10 cm cell culture dishes. After cell adherence, the cells were treated with the drug (0, 2.5, 5, and 10 μM) for 24 h. After 24 h, the cells were washed with PBS three times and collected by trypsin. The number of collected cells was counted by a cell counter. Then, the cells were digested in 1 mL HNO_3_ solution by microwave digestion at 120 °C for 3 min, and 1 mL of this solution was transferred to a 5 mL volumetric flask, diluted with distilled water to volume, and mixed. The Ru content of these solutions was analyzed by an ICP optical emission spectrometer (NCS, Plasma 2000). All measurements were repeated three times.

### 3.6. Molecular Docking

Molecules were optimized using the Amsterdam density function 2019. The initial PDB structures of the optimized Ru complexes were created using Mercury software. AutoDock4.2 software was used to calculate the binding mode and energy–site interaction properties of the compounds with c-myc G4 DNA by the Lamarckian genetic algorithm [42]. The theoretical calculation sequence of c-myc G4 DNA (PDB ID: 2L7V) was obtained from the protein database. For all nucleotide sequences, the area of the calculation box was 126 × 126 × 126, and the center point was 1.000. A total of 100 individual joints were examined, and the calculated binding energy was higher than 2.5 × 10^7^ kcal·mol^−1^. Finally, the conformation with the lowest binding energy was selected as the best conformation with the maximum potential energy.

### 3.7. UV Titration

The affinity between c-myc G4 DNA and the complexes was determined by electron absorption spectrometry at room temperature [43]. First, 3.0 mL Tris-HCl–KCl buffer solution (10 mM Tris-HCl, 100 mM KCl, pH 7.4) was used as a reference, and the spectrum in the range of 200–800 nm was recorded. In UV–Vis absorption spectrometry, a sample solution of 20 μM was obtained by placing 6 μL of the complex in the sample cell, and the same volume of drug solvent was placed in the reference dish. A microinjector was used to add 2 μL DNA solution to increase the concentration ratio of DNA and complex in a certain proportion. The test was started after stabilization for 2 min each time, and changes in the electronic absorption spectra of the complex were monitored in the range of 200–800 nm. The binding constant was calculated using the following equation:

(ε_a_ − ε_f_)/(ε_b_− ε_f_) = [b − (b^2^ − 2K^2^C_t_[DNA]/S)]^1/2^/2KC_t_, (1)

B = 1 + KC_t_ + K[DNA]/2S (2)

where [DNA] is the free DNA concentration, K_b_ is the apparent binding constant, ε_a_ corresponds to the extinction coefficient observed, and ε_f_ corresponds to the extinction coefficient of the free compound. ε_b_ is the extinction coefficient of the compound when fully bound to DNA. C_t_ is the total metal complex concentration, and S is the binding size.

### 3.8. FID Assay

The TO replacement test was performed by adding complexes ***Λ***−**3** and ***Δ***−**3** with gradually increasing concentrations of the mixture containing c-myc G4 DNA-TO (1.0 μM c-myc G4 DNA + 0.5 μM TO). After allowing the mixture to stand for 2 min, the emission spectrum was recorded [33]. The TO replacement rate was calculated using the TO replacement rate equation (%) = 100 − (F_t_/F_0_) × 100, where F_t_ is the fluorescence intensity of each titration point and F_0_ is the fluorescence intensity of c-myc G4 DNA–TO binding. Origin software was used to analyze the data and determine the DC_50_ value in accordance with the relationship between TO displacement (%) and the complex concentration.

### 3.9. CD Spectrum

A quartz colorimetric dish with a diameter of 1 cm was used to detect the CD spectra. The wavelengths of spectrum acquisition were 200–600 nm, the bandwidth was 1.0 nm, and the response time was 0.5 s. Different concentrations of c-myc G4 DNA were added into Tris-HCl buffer with the complex (20 μM). After each addition of DNA and stirring and balancing for at least 2 min, the CD spectra were scanned and collected. The baseline was collected in the same colorimetric dish and subtracted from the spectrum, subtracting the background. OriginPro 9.0 software (Origin Lab Corp. Northampton, America) was used for data analysis.

### 3.10. FRET assay

Carboxy fluorescein (FAM) and 6-carboxytetramethyl rhodamine (TAMRA) double fluorescent-labeled c-myc G4 DNA (5′−FAM-TGAGGGTGGGAGGGGTAA-TAMRA−3′) were heated to 95 °C in Tris-HCl-KCl buffer (containing 10 mM Tris-HCl, 100 mM KCl, pH 7.4) for 5 min and slowly cooled to room temperature. To ds26, double-stranded DNA (CAATCGATCGAATTCGATCCGATTG) is a competitive binder, research ***Λ/Δ*−3** with c-myc G4 selectivity binding ability of DNA. A Bio-Radiq5 real-time PCR detection system was used to measure the fluorescence melting curve. Approximately 1 μM (5 μL) FPU22T was mixed with a series of 5 μL compound solutions of different concentrations, and 15 μLTris-HCl-KCl buffer was added to the PCR 8 tube to obtain a total volume of 25 μL. A real-time fluorescence quantitative PCR instrument was used, and incubation was performed at 30 °C for 30 min. The program temperature range was set to 30–95 °C at a temperature interval of 1 °C, and recording was performed after balancing for 30 s to detect the fluorescence value of FAM. The conditions of the reaction system were similar to those of the FRET melting point test except that different concentrations of ds26 were added.

### 3.11. PCR-Stop Assay

In this study, c-myc G4 DNA and its reverse complementary sequence c-myc rev were heated to 95 °C for 5 min, allowed to cool to room temperature, and incubated at 4 °C overnight. The system was supplemented with dNTPs and Taq polymerase for PCR amplification; heated at 95 °C for 3 min, 94 °C for 30 s, and 58 °C for 30 s, with an extension at 72 °C for 30 s (30 cycles); and analyzed by 15% nondenatured polyacrylamide gel electrophoresis and silver staining.

### 3.12. Wound-Healing Assay

MDA-MB-231 cells (1 × 10^5^ cells/well) were inoculated in a 6-well cell culture plate marked on the back until a single layer of cells covered more than 90% of the bottom of the culture plate, and a 200 µL pipette was used to scratch the culture. Different concentrations of the complexes (0, 2.5, 5, and 10 µM) were applied for 72 h. Migrating cells in the same field of view were observed and recorded every 24 h, and the average cell migration rate was calculated in three fields for every group.

### 3.13. FITC–Gelatine Assay

An appropriate amount of FITC–gelatine glue was pipetted to cover the inner ring of the confocal cuvette, added to 0.5% glutaraldehyde for at least 30 min after solidification, and activated with 5 mg·mL^−1^ sodium borohydride. Subsequently, 2 mL of cell suspension (5 × 10^4^ cells) treated with different drug concentrations (0, 2.5, 5, and 10 µM) was added to each dish. The mixture was incubated at 37 °C for 24 h, and then the cells in each dish were fixed with 4% paraformaldehyde, incubated with phalloidin staining solution for 30 min and DAPI for 5 min, and imaged with a fluorescence microscope in the dark.

### 3.14. Immunofluorescence

MDA-MB-231 cells were inoculated in a 6-well plate containing a cover glass. On the second day, the cells were treated with different concentrations of the complex (0, 5, and 10 μM) for 24 h. The cells were fixed with 4% paraformaldehyde for 20 min, and then blocked with 5% BSA for 0.5 h at room temperature. The 1:500 anti-γH2AX antibody was diluted with 5% FBS and incubated for 1 h. The cells were incubated with lgG (H + L) for 0.5 h and treated with DAPI for 5 min. The cover glass was then transferred to a specified microscope slide and photographed with laser confocal microscopy. The antibodies used in immunofluorescence to determine invasion and metastasis were anti-paxillin antibody (ab32115), Fluor 488-conjugated IgG (H + L) (Proteintech), and rhodamine phalloidin (Amanita phalloidin, cytoskeleton).

### 3.15. Flow Cytometry

MDA-MB-231 cells were treated with different concentrations of the complexes (0, 10, 20, and 40 μM) for 72 h, then collected and stained with an Annexin V-FITC/PI Apoptosis Detection Kit I (BD, 556547). The MDA-MB-231 cells were fixed with 70% ethanol at 4 °C for 30 min, and then collected and stained with PI/RNase Staining Buffer (BD, 550825). The cells were analyzed by flow cytometry (Beckman, CytoFLEX). All experiments were repeated three times.

### 3.16. Comet Assay

DNA damage was detected by single-cell gel electrophoresis by using the Comet assay kit (Trevigen) [44]. MDA-MB-231 cells were added to a culture dish containing the medium. After treatment with different concentrations of the complexes (0, 5, and 10 μM) at 37 °C for 72 h, the cells were collected and washed with PBS three times. A solution of the cell suspension and melted low-melting-point agarose gel was mixed at a volume ratio of 1:7, and the equilateral sample was immediately transferred to a Comet Slide TM slide. The slides were washed twice with PBS, placed into an electrophoresis tank containing comet electrophoresis solution, and electrophoresed at 25 V for 30 min. After electrophoresis, the slides were washed twice with PBS. Finally, the cells were stained with EB and observed under a fluorescence microscope. Cells were randomly selected from each slide to take photos, and the tail moment was analyzed.

### 3.17. In Vivo Activity Evaluation using the Zebrafish Xenograft Model

The zebrafish model of transplanted breast cancer was constructed in accordance with the methods in the literature [39]. Juvenile 48 h old breast cancer model zebrafish were placed in 48-well plates (n = 10), and 0.2 mL aquaculture water without or with ***Δ***−**3** (0, 2.5, 5, and 10 μM) was added for culture. Images were captured every 24 h using a fluorescence microscope to study the inhibitory effect of ***Δ***−**3** on zebrafish with breast cancer. In addition, the mean number of sprout and mean length of sprout in subintestinal vessels (SIVs) were analyzed by ImageProPlus software. The ethical protocols for the in vivo research on zebrafish complied with relevant laws.

### 3.18. Tube Formation Assay

Dissolving matrigel (4 °C, 24 h) was used to coat prechilled 96-well plates (50 μL/well, 37 °C, for at least 60 min). Then, the unsolid fluid was removed, and HUVECs at the density of 3 × 10^4^ were cultured in DMEM with VEGF (200 ng/mL) containing the different concentrations of ***Δ***−**3** (0, 5, and 10 μM) for 6 h. These data were analyzed by ImageProPlus software.

## 4. Conclusions

A class of DPPZ-based Ru(II) complexes with different modifications was synthesized under microwave irradiation. Analysis of the structure–activity relationships of the synthetic complexes revealed that the introduction of a 3-aminophenylethynyl group promoted the entry of DPPZ-based Ru(II) complexes into the cell nucleus and enhanced cytotoxicity. Further analysis indicated that the dextro-isomer of the DPPZ-based Ru(II) complex containing a 3-aminophenylethynyl group more easily entered the cell nucleus and exhibited better inhibition than the levo-isomer. The ***Δ***−**3** complex could recognize and stabilize the c-myc G-quadruplex through certain intermolecular H bonding and π–π stacking interactions, resulting in the suppression of transcription and expression of c-myc. The ***Δ***−−***3*** complex displayed a prominent inhibitory effect against the proliferation, migration, and invasion of MDA-MB-231 cells through DNA-damage-mediated apoptosis. Moreover, the assessment of in vivo activity demonstrated that ***Δ***−**3** blocked the growth of MDA-MB-231 cells in the zebrafish xenograft model and inhibited angiogenesis in vitro and in vivo. These discoveries encourage the introduction of the phenylethynyl group into DPPZ-based Ru(II) complexes to enhance cytotoxicity and highlight their potential for use as c-myc G-quadruplex DNA binders.

## Figures and Tables

**Table 1:** Inhibitory effect (IC_50_/μM) of chiral DPPZ-based Ru(II) complexes and cis-platin against various human tumor and normal cell lines.

Comp.	Inhibitory Effect (IC_50_/μM)						Selectivity Index *
	MDA-MB-231	MCF-7	HepG2	A549	CNE-1	HaCaT	
***Λ***−**1**	60.81 ± 7.41	>100	>100	>100	>100	>100	/
***Δ***−**1**	>100	>100	>100	>100	>100	>100	/
***Λ***−**2**	92.06 ± 12.09	>100	>100	91.47 ± 10.81	>100	>100	/
***Δ***−**2**	94.50 ± 9.10	>100	>100	89.87 ± 11.35	>100	>100	/
***Λ***−**3**	53.5 ± 1.7	146.9 ± 2.7	74.8 ± 2.4	55.4 ± 2.4	>100	>100	/
***Δ***−**3**	25.51 ± 1.42	90.64 ± 6.34	37.78 ± 0.6	24.66 ± 0.28	40.94 ± 0.53	70.95 ± 6.81	2.78
Cis-platin	25.9 ± 1.9	14.27 ± 0.07	12.68 ± 0.7	9.90 ± 0.25	/	7.1 ± 0.5	0.27

* Selectivity Index = IC_50_ (HaCaT)/IC_50_(MDA-MB-231).

## Data Availability

The data presented in this study are openly available in the article and Supplementary Materials.

## Supplementary Materials

The following supporting information can be downloaded at: https://www.mdpi.com/article/10.3390/ijms24010203/s1.
