# Supplementary material for: Novel Chiral Ru(II) Complexes as Potential c-myc G-quadruplex DNA Stabilizers Inducing DNA Damage to Suppress Triple-Negative Breast Cancer Progression
Source: Int J Mol Sci. 2022 Dec 22;24(1):203. doi: 10.3390/ijms24010203 (PMC9820592; doi:10.3390/ijms24010203)
Supplement: Supplementary file 1 [file ijms-24-00203-s001.zip › ijms-2040214-supplementary.pdf]

Supplementary Materials

for

Novel Chiral Ru(II) Complexes as Potential c-myc G-quadruplex DNA Stabilizers Inducing DNA Damage to Suppress Triple-Negative Breast Cancer Progression

Chanling Yuan [1], Zhixiang Wang [1], Zongtao Wang [1], Wentao Liu [1], Guohu Li [1], Jinlan Meng [2], Ruzhen Wu [1], Qiong Wu [3,4,*], Jiacheng Wang [1] and Wenjie Mei [1,4,*]

[1] School of Pharmacy, Guangdong Pharmaceutical University, Guangzhou 510006, China
[2] Department of Physiology, School of Life Sciences and Biopharmaceutics, Guangdong Pharmaceutical University, Guangzhou 510006, China
[3] Institute of Biological and Medical Engineering, Guangdong Academy of Sciences, Guangzhou 530316, China
[4] Guangdong Engineering Technology Research Centre of Molecular Probe and Biomedicine Imaging, Guangdong Pharmaceutical University, Guangzhou 510006, China
* Correspondence: wuqiongniu.1113@163.com (Q.W.); wenjiemei@gdpu.edu.cn (W.M.)

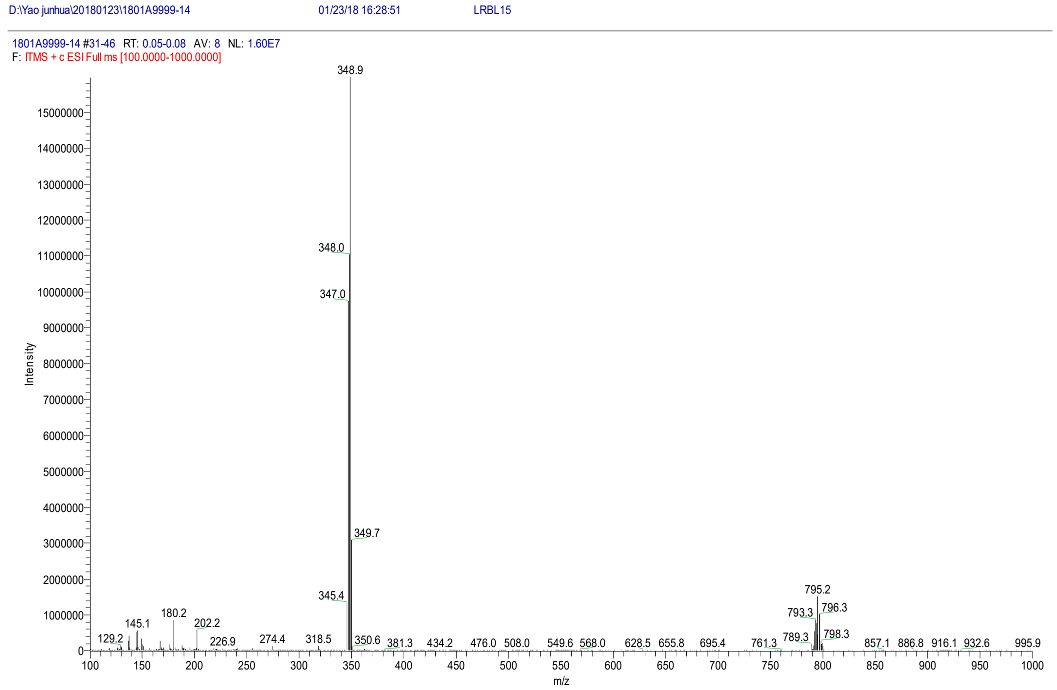

Figure S1. The ESI-MS spectra of ***Λ*-1**.

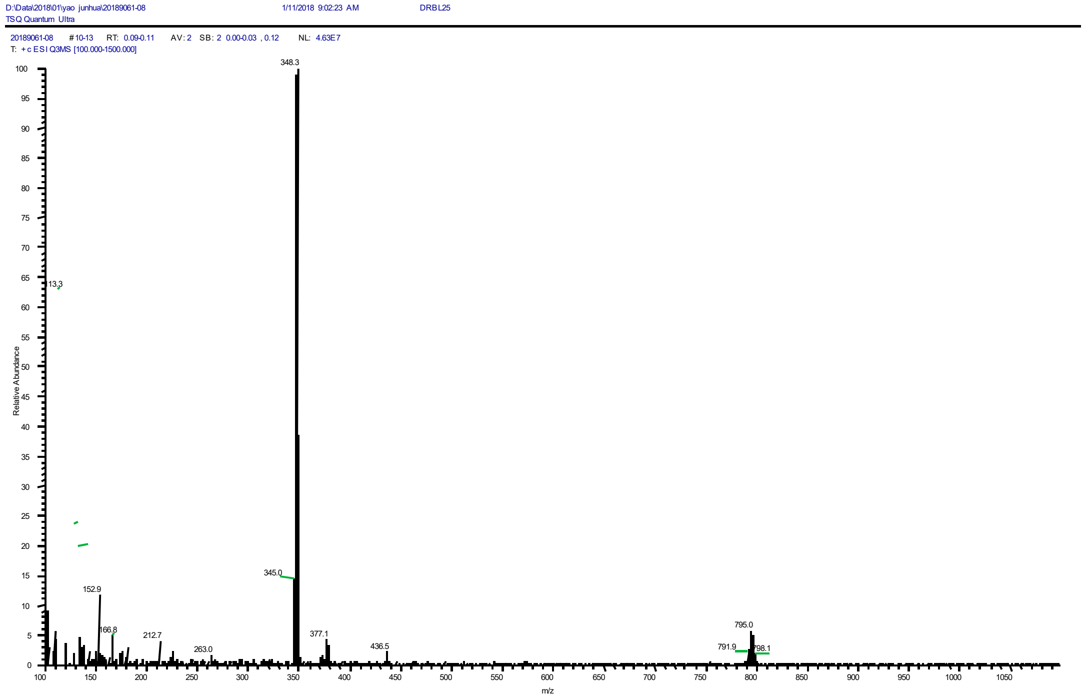

Figure S2. The ESI-MS spectra of ***Δ*-1**.

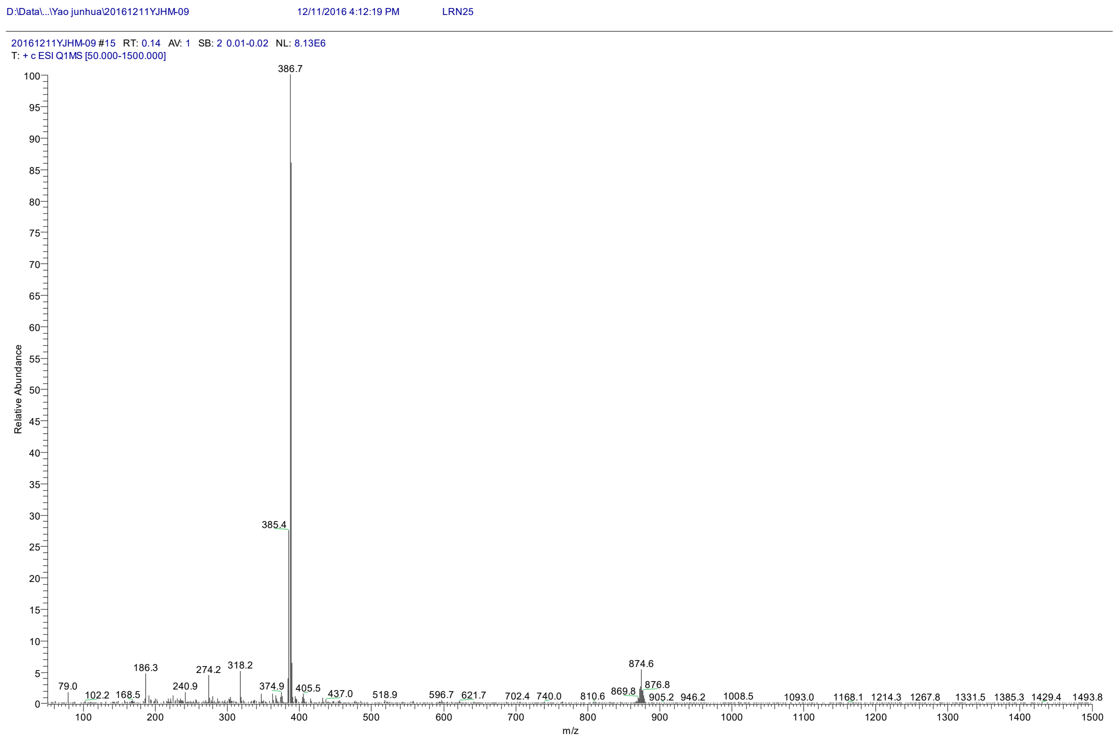

Figure S3. The ESI-MS spectra of ***Λ*-2**.

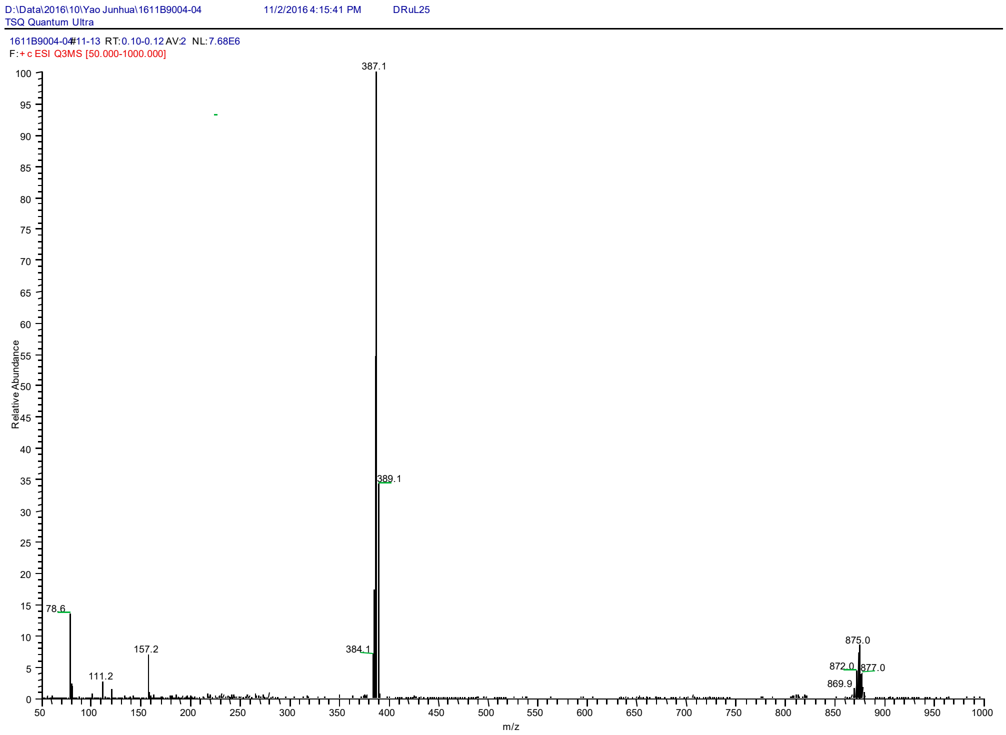

Figure S4. The ESI-MS spectra of ***Δ*-2**.

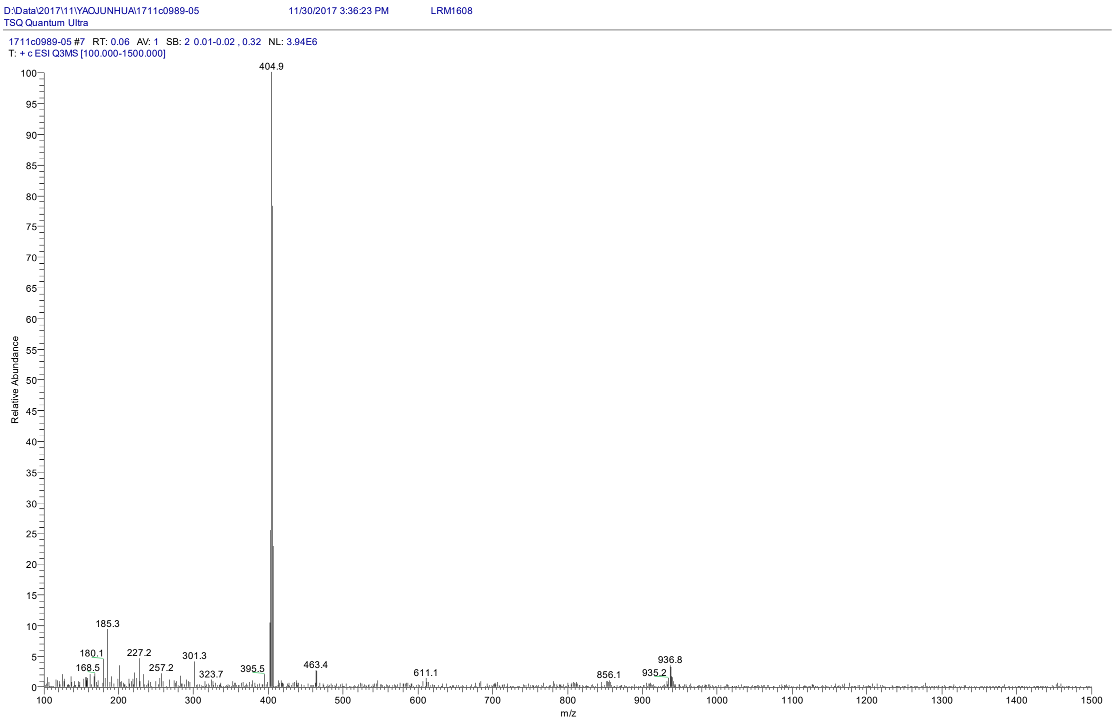

Figure S5. The ESI-MS spectra of ***Λ*-3**.

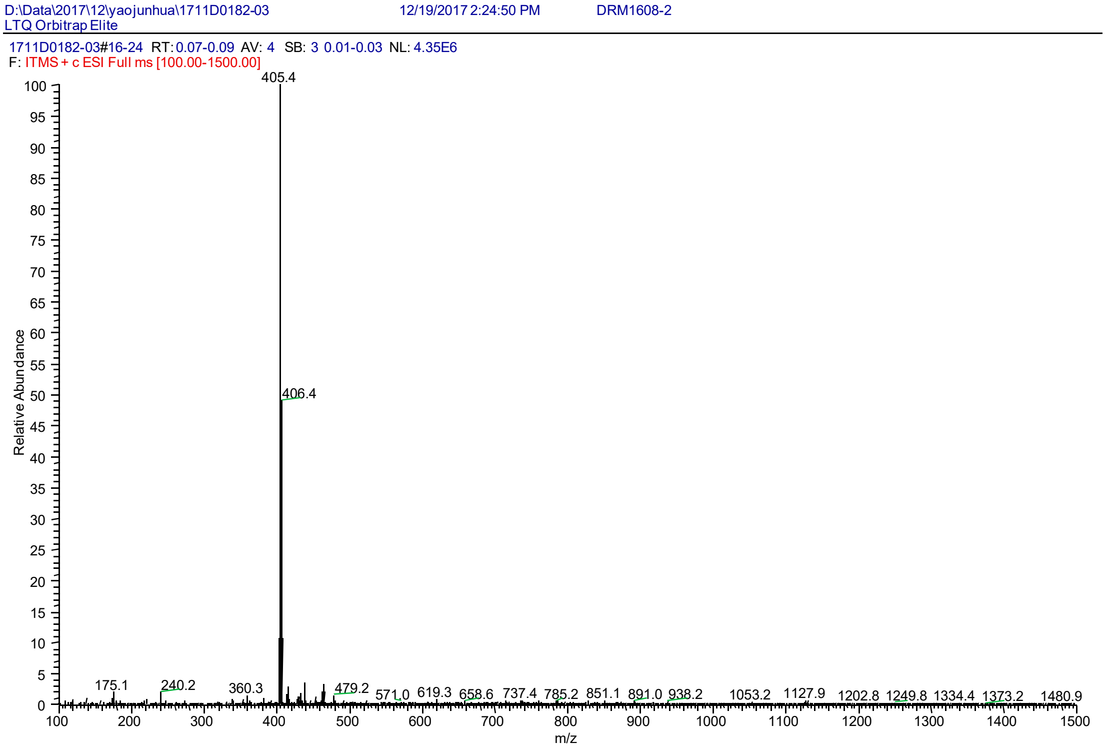

Figure S6. The ESI-MS spectra of ***Δ*-3**.

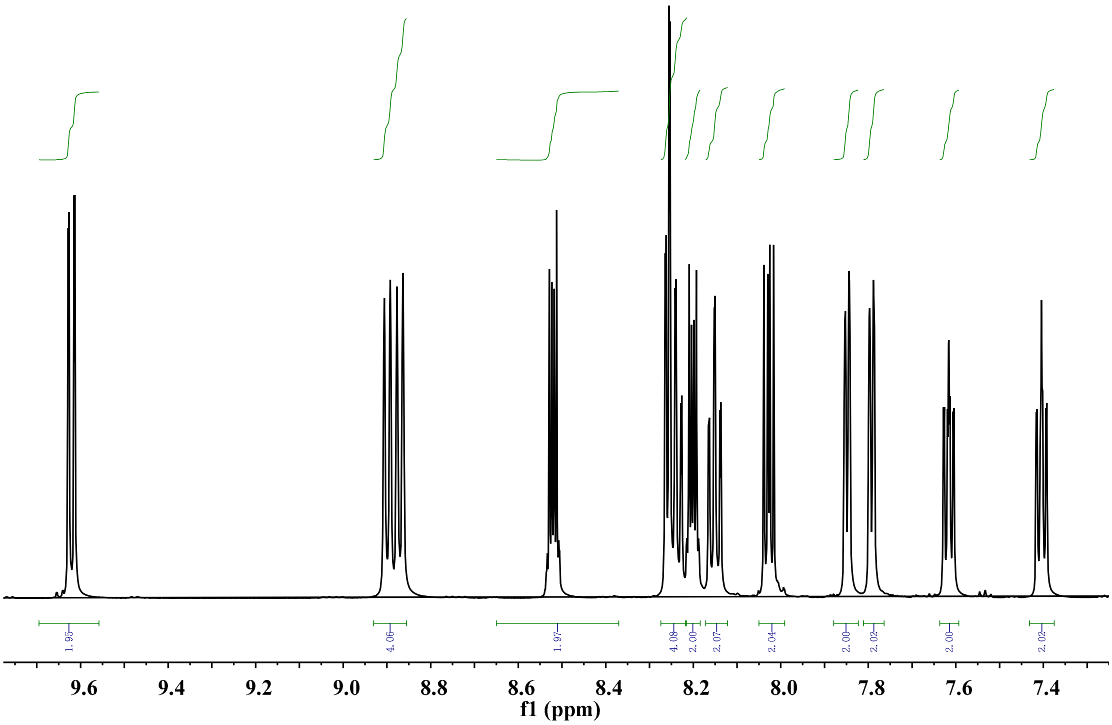

Figure S7. The ^{1}H NMR spectra of ***Λ*-1**.

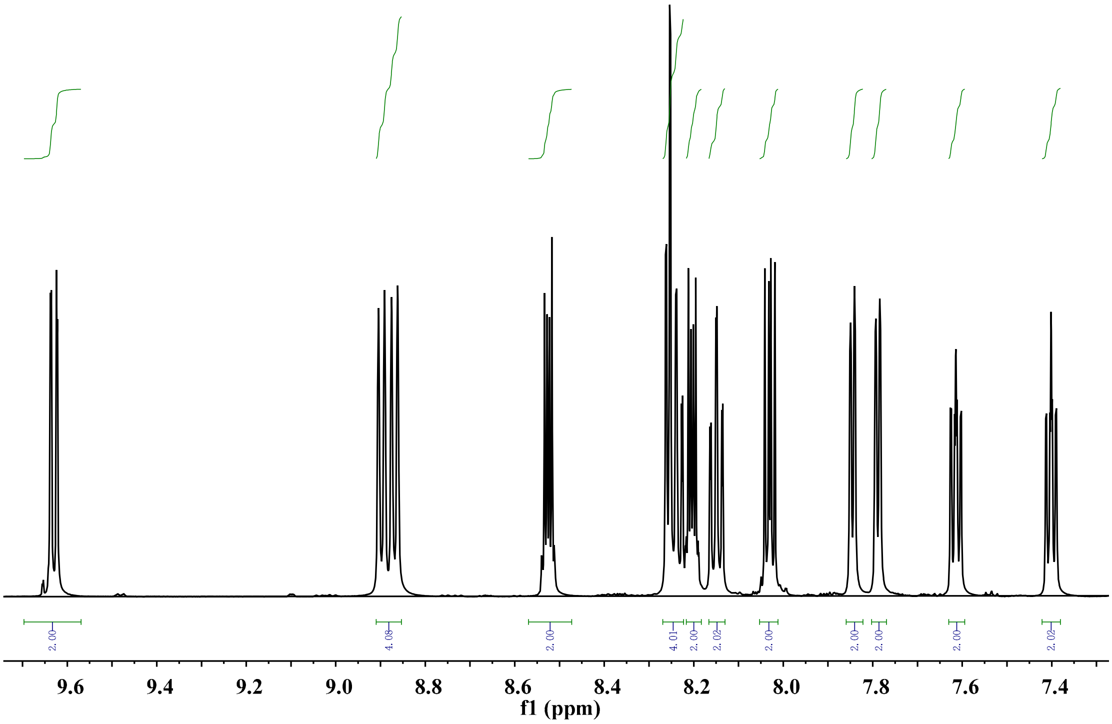

Figure S8. The ^{1}H NMR spectra of ***Δ*-1**.

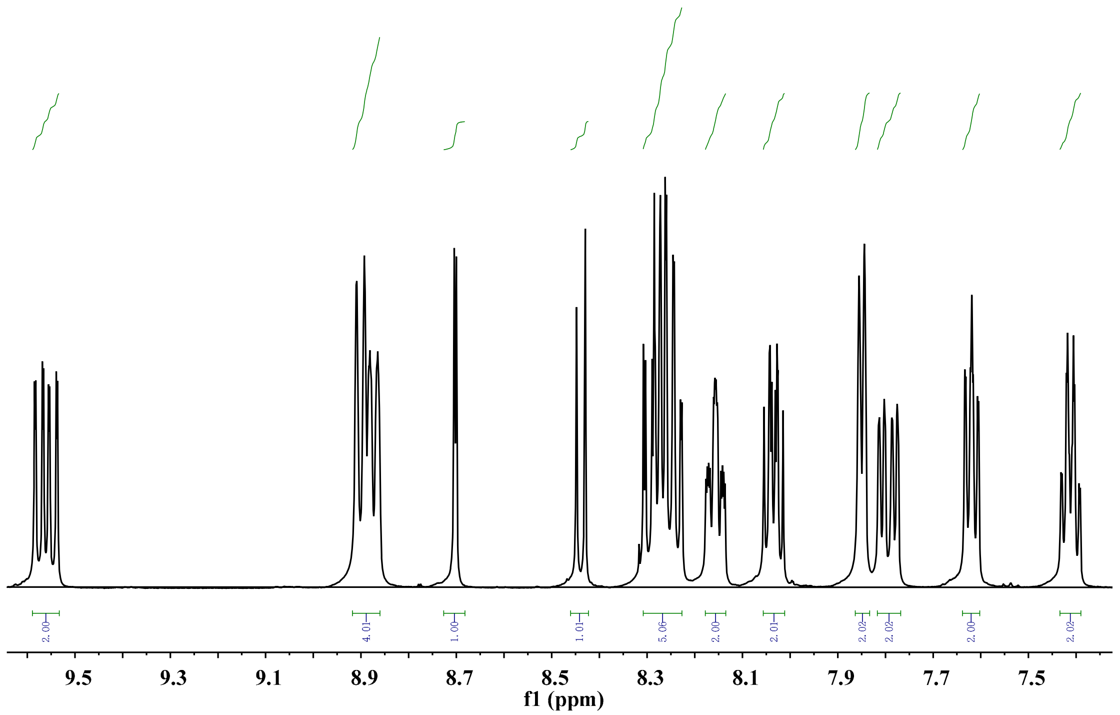

Figure S9. The ^{1}H NMR spectra of ***Λ*-2**.

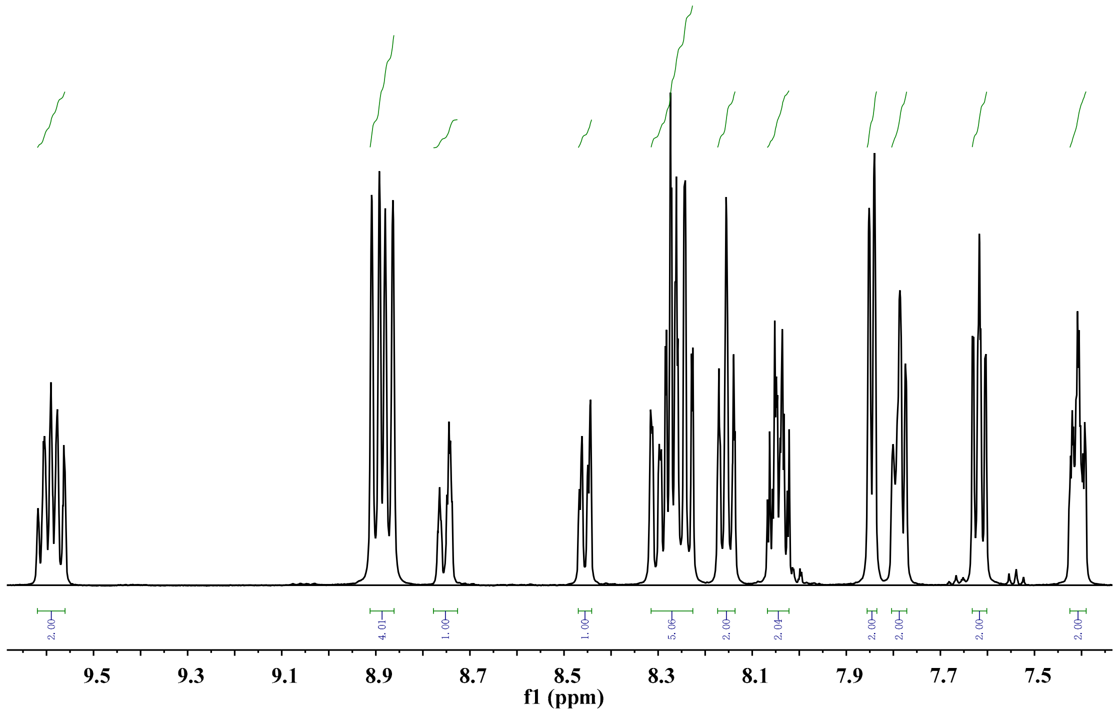

Figure S10. The ^{1}H NMR spectra of ***Δ*-2**.

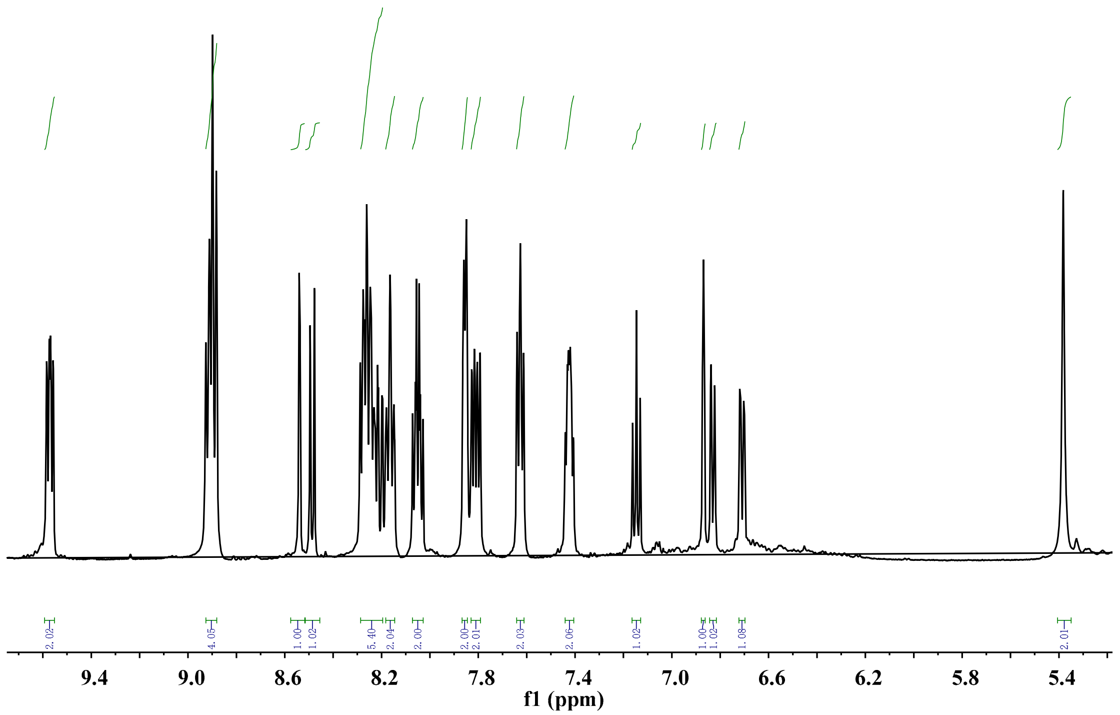

Figure S11. The ^{1}H NMR spectra of ***Λ*-3**.

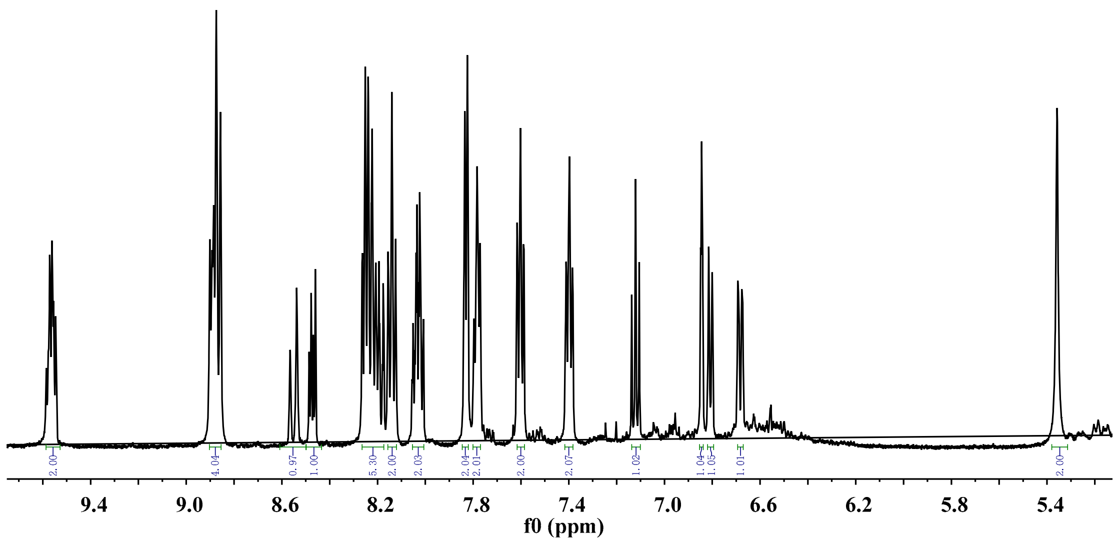

Figure S12. The ^{1}H NMR spectra of ***Δ*-3**.

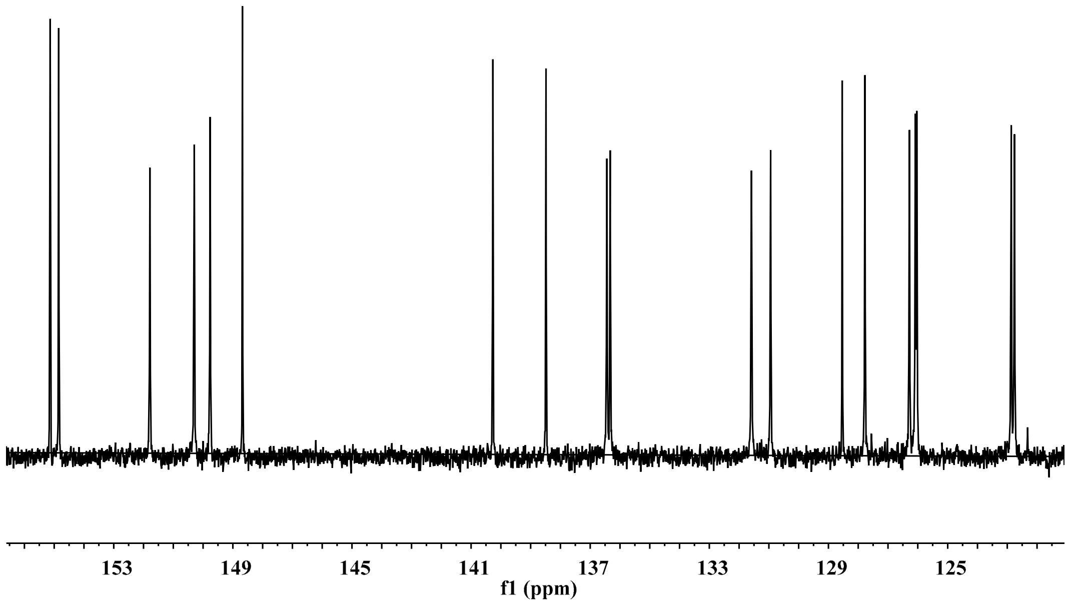

Figure S13. The ^{13}C NMR spectra of ***Λ*-1**.

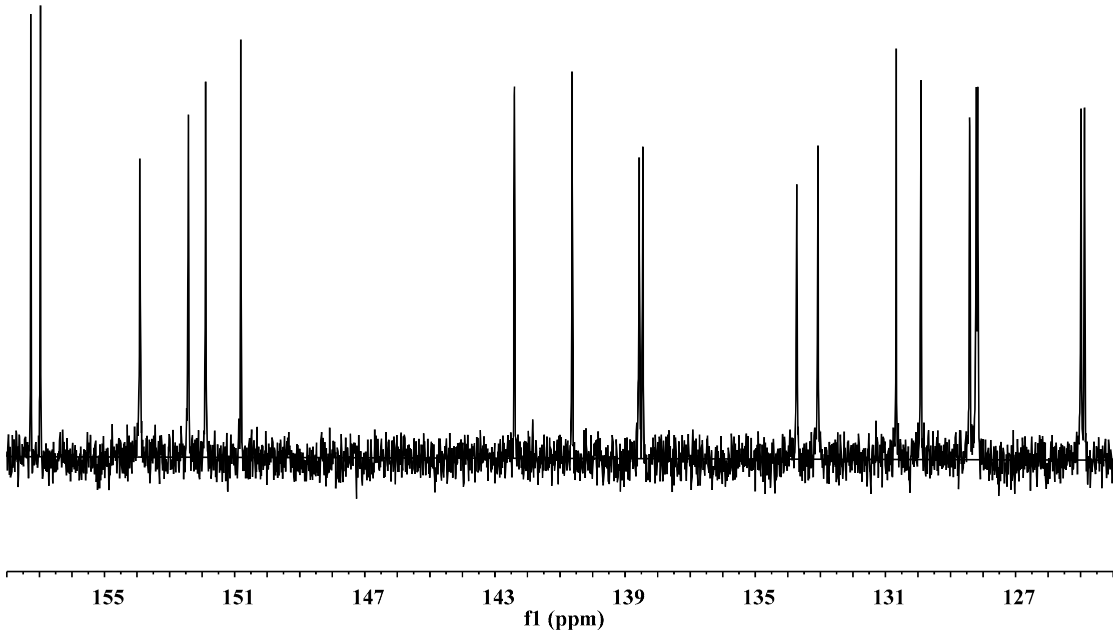

Figure S14. The ^{13}C NMR spectra of ***Δ*-1**.

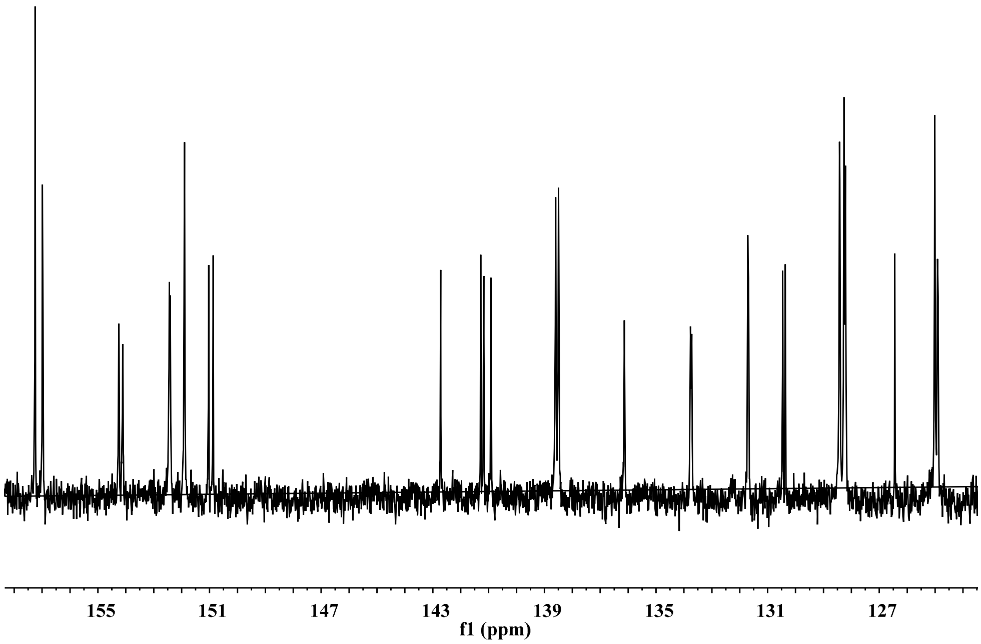

Figure S15. The ^{13}C NMR spectra of *Λ*-**2**.

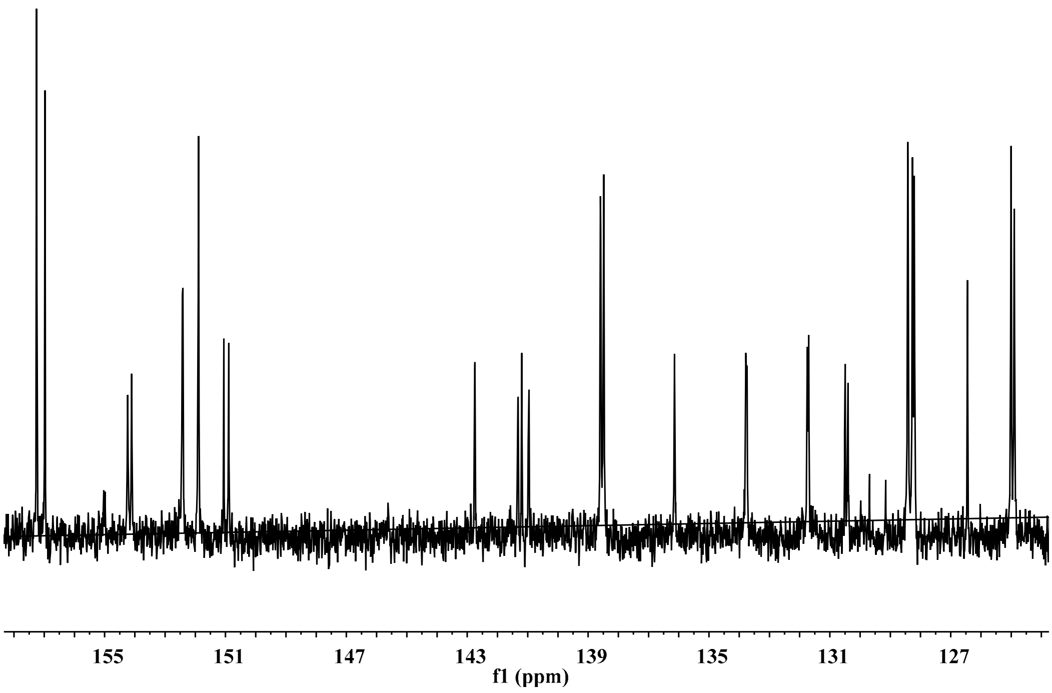

Figure S16. The ^{13}C NMR spectra of *Δ*-**2**.

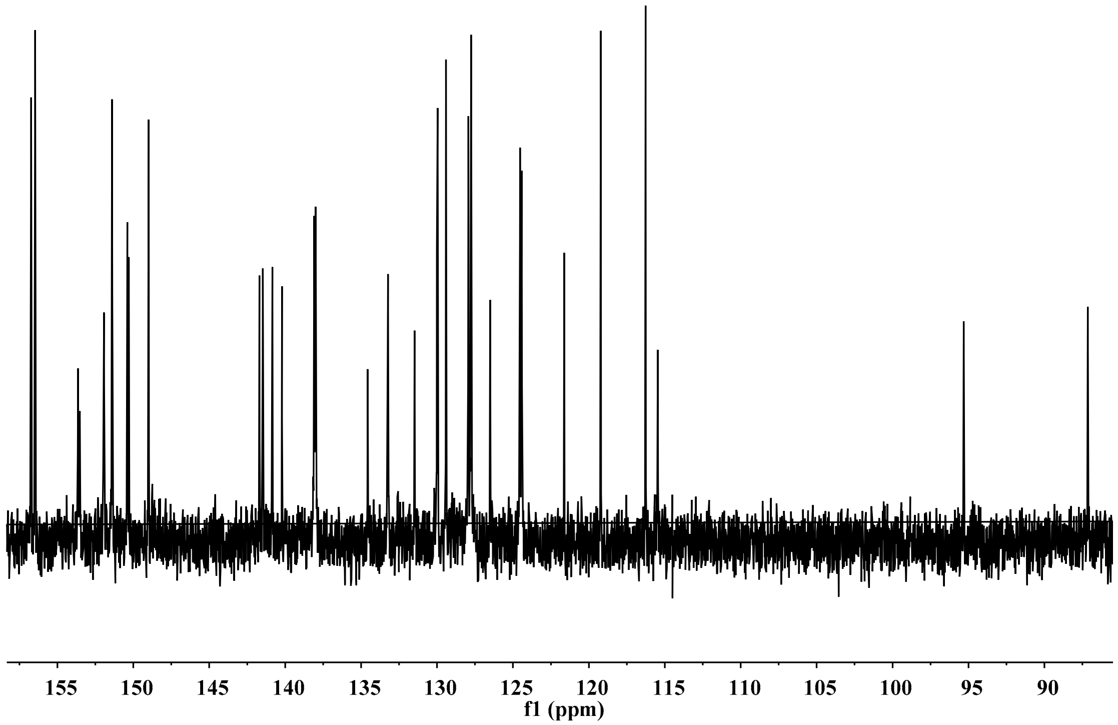

Figure S17. The ^{13}C NMR spectra of *Λ*-**3**.

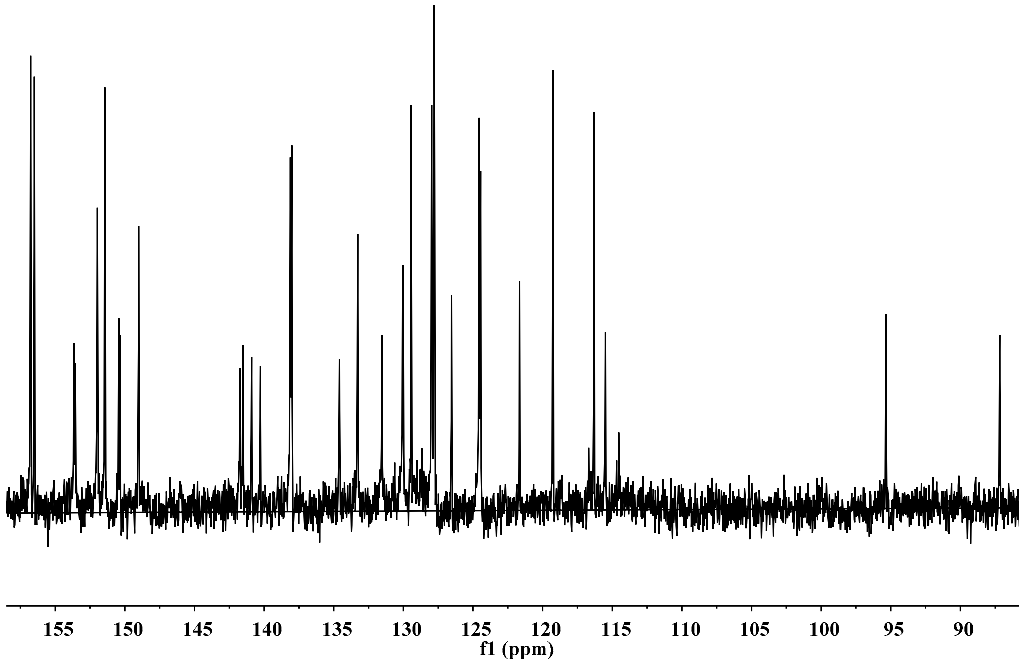

Figure S18. The ^{13}C NMR spectra of *Δ*-**3**.

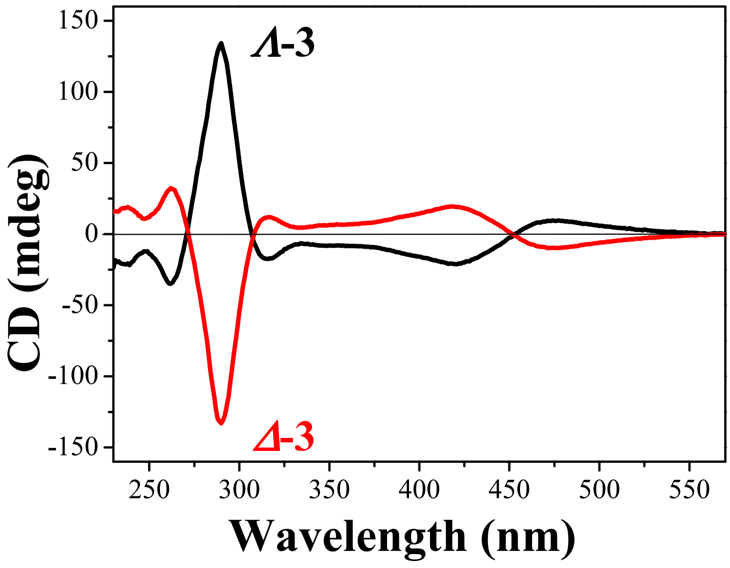

Figure S19. The CD spectra of ***Λ*-3** and ***Δ*-3**.

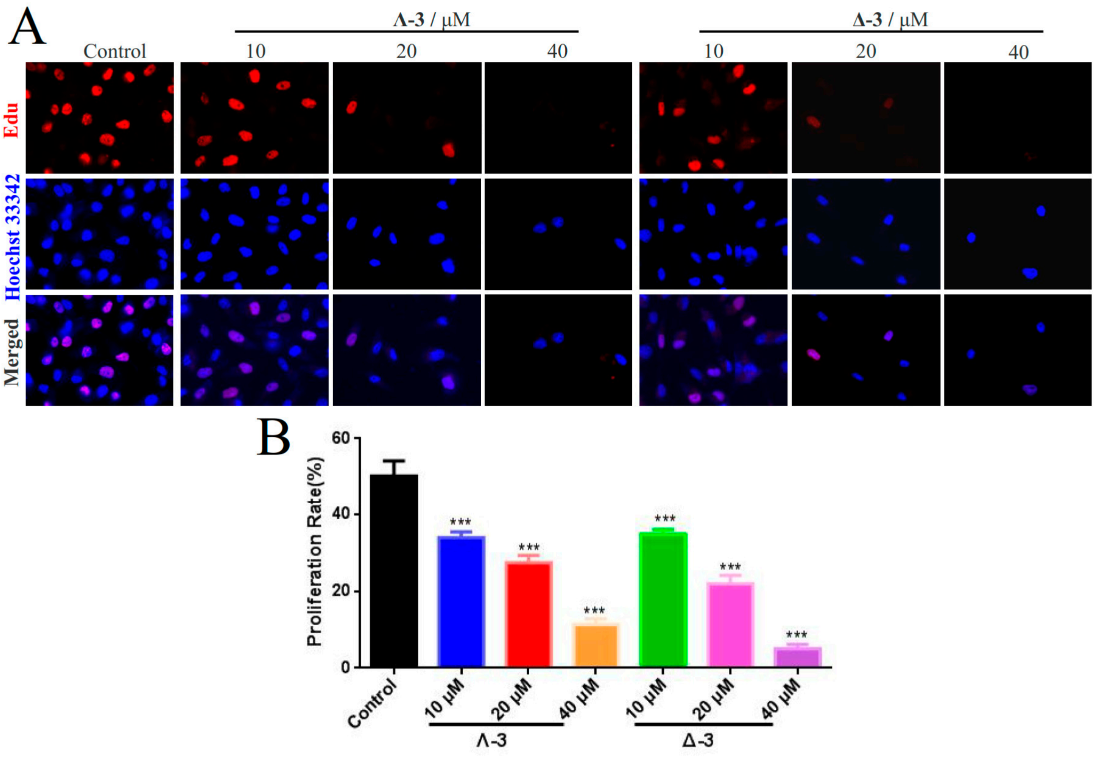

Figure S20. (A) EdU proliferation assay analysis and (B) proliferation rate of the effect of ***Λ*-3** and ***Δ*-3** on the growth of MDA-MB-231 cells.

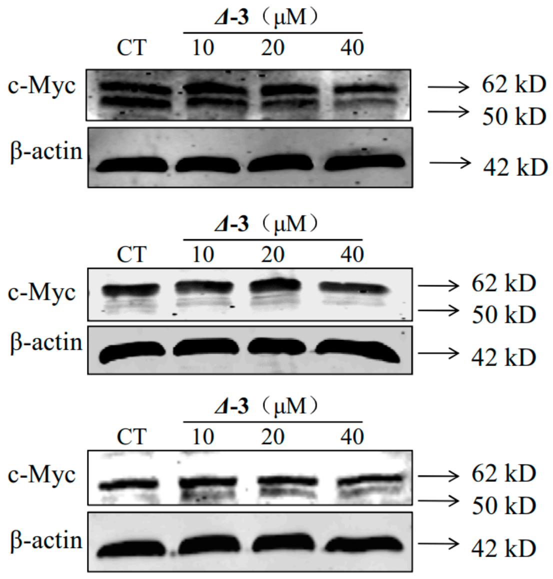

Figure S21. The expression of c-myc protein changed after dealt with ***Δ-3*** (0, 10, 20 and 40 μM) in MDA-MB 231 cells for 72 h (three repeat measurement).

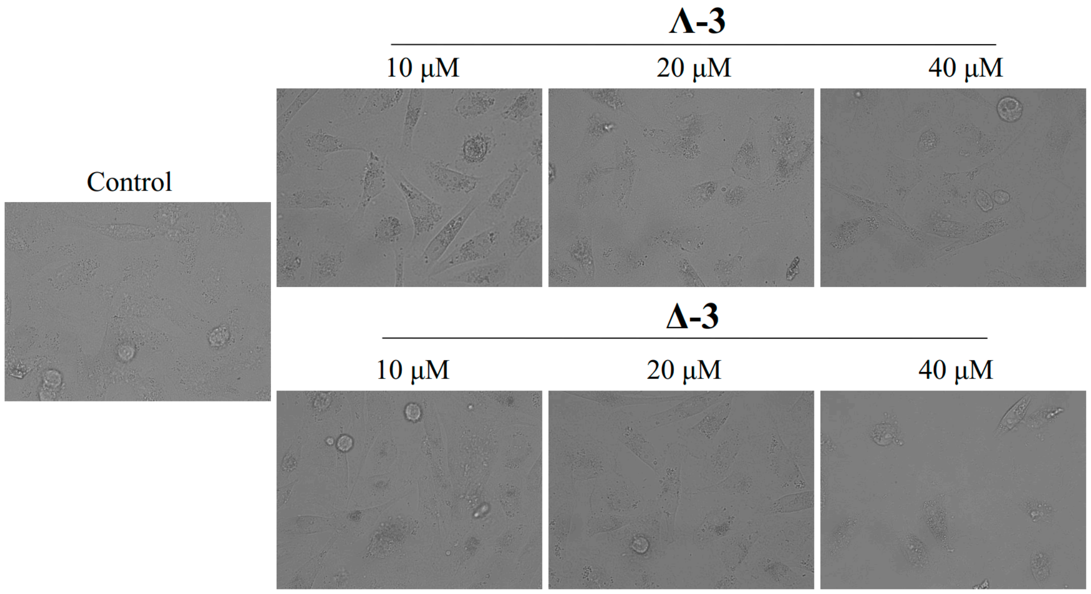

Figure S22. The morphological change of MDA-MB-231 cells in the absence and presence of ***Λ*-3** and ***Δ*-3** (0,10, 20 and 40 μM) for 72 h.

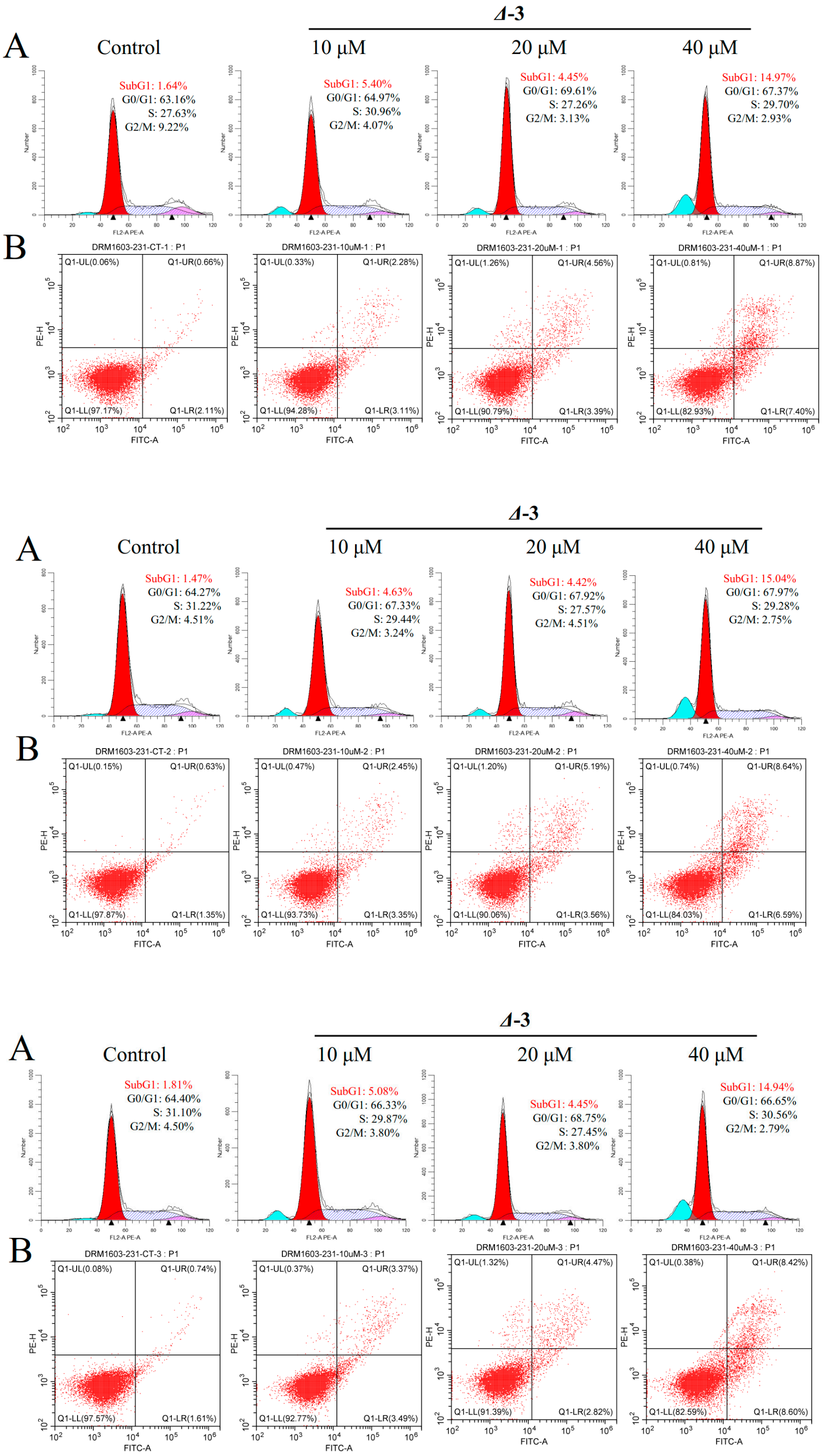

Figure S23. Cell cycle distribution (A) and apoptosis (B) of MDA-MB-231 cells incubated with ***Δ*-3** (0, 10, 20 and 40μM) for 72 h tested by flow cytometric analysis (three biological repetition).

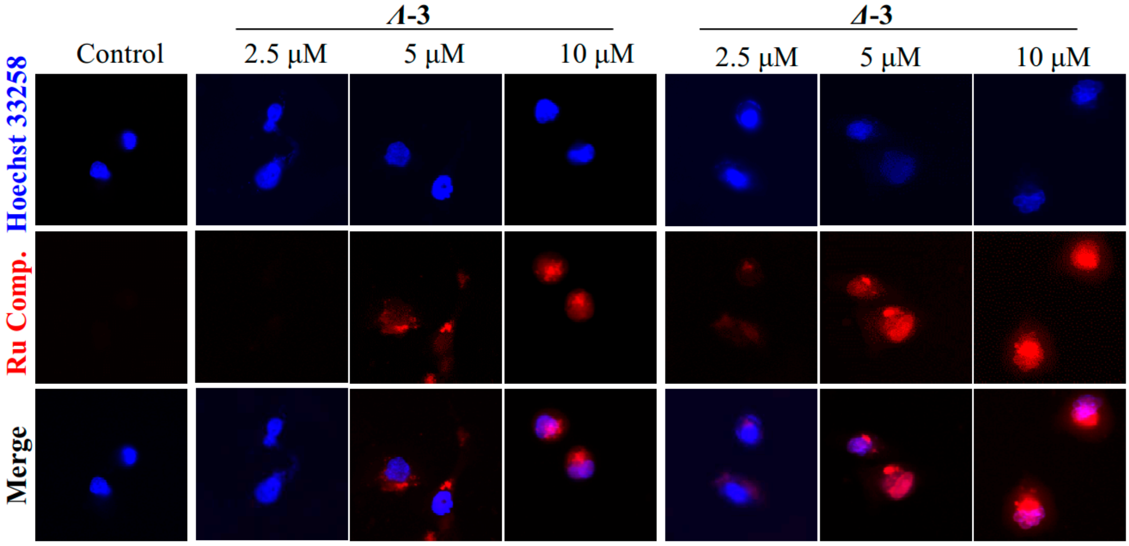

Figure S24. The cellular localization of ***Λ*-3** and ***Δ*-3** in MDA-MB-231 cells. MDA-MB-231 cells treated with ***Λ*-3** and ***Δ*-3** (2.5, 5 and 10 μM) for 24 h. Red: Ru(II) complex, blue: Hoechst33258.
